# Spatiotemporal evolution of seismicity during the cyclic operation of the Hutubi underground gas storage, Xinjiang, China

**DOI:** 10.1038/s41598-022-18508-x

**Published:** 2022-08-24

**Authors:** Bo Zhang, Baoshan Wang, Bin Wei, Zhide Wu, Ni-Er Wu, Renqi Lu, Zhanbo Ji, Jinxin Hou, Lu Li

**Affiliations:** 1 Institute of Geophysics, China Earthquake Administration, Beijing, China; 2 School of Earth and Space Sciences, University of Science and Technology of China, Hefei, China; 3 Mengcheng National Geophysical Observatory, University of Science and Technology of China, Hefei, China; 4 Earthquake Agency of Xinjiang Uygur Autonomous Region, Urumqi, China; 5 Research Institute of Petroleum Exploration and Development, Langfang, China; 6 Institute of Geology, China Earthquake Administration, Beijing, China; 7 Chinese Academy of Geological Sciences, Beijing, China

**Keywords:** Geophysics, Natural hazards, Seismology

## Abstract

Underground gas storages (UGSs) are important large-scale industrial facilities used to bridge the gap between natural gas consumption and supply. The cyclic operation of the UGS may alter the subsurface stresses and local seismicity. We examined seismicity around the Hutubi UGS from 2011 to 2019 using the matched filter technique (MFT) and double-difference location methods. More than 1300 earthquakes were detected with seismicity around the UGS showing a remarkable increase since the start of its operation and showing a clear correlation to seasonal gas production. About 684 detected earthquakes were located, most of them occurred within 6 km of the reservoir. The events can be grouped into two clusters. Both clusters initiated around the gas pressure boundary. The first cluster extinct after the first injection period. While the second cluster diffused upward along a pre-existing fault. We speculate that strain localization caused by non-uniform gas injection contributes to the initiation of seismicity clusters around the UGS, and the trapped crude oil/gas played an important role in the migration of the second surge. The revealed seismicity pattern contributes to a better understanding of the mechanism of induced seismic events and emphasizes the importance of seismic monitoring in the UGS region.

## Introduction

In recent years, the incidence of earthquakes associated with anthropogenic activities has gained increasing attention from both the scientific community and the general public^1,2^. It has been reported that earthquakes can be induced by large-scale industrial activities, such as the exploitation of oil or underground water^3^, unconventional hydrocarbon development^4–6^, and geothermal exploitation^7,8^. In such cases, induced seismicity is often related to the injection and extraction of underground liquids. Moreover, industrial activities related to underground gas operations, such as CO_2_ storage^9,10^, natural gas extraction^11,12^, and underground gas storage (UGS)^13–16^ may also induce earthquakes.

Globally, the construction of UGSs is increasing to bridge the gap between natural gas consumption and supply^17^. The natural gas consumption shows clear seasonality, but the production does not. To balance the consumption and production, the natural gas is injected into the UGS during periods of low demand, and extracted from the UGS to meet high demand. The construction and operation of UGSs may cause subsurface stress perturbations^18–20^, which can alter regional seismicity patterns^13–16^.

In contrast to hydraulic fracturing and natural gas extraction, UGS operation is accompanied by persistent cyclical loading and unloading. It is understood that this repeated injection and extraction process may change local seismic hazards, which have implications for the safe operation of UGSs^2^. However, earthquakes related to the operation of gas storage facilities are less reported.

In June 2013, the largest UGS in China was opened in Hutubi, Xinjiang^21^. The Hutubi area is well equipped with different geophysical measuring instruments, which makes this region an ideal place for investigating the spatiotemporal evolution of seismicity related to UGS operation. Seismicity in the Hutubi area from 2013 to 2015 shows some correlation with UGS production^16,22^. However, no consensus has been reached as to the mechanism responsible for these changes^16,18,19,22^. In addition, the impact of long-term cyclical UGS operation on seismicity requires further investigation.

In this study, we examine the effect of the Hutubi UGS on local seismicity between 2011 and 2019, including six complete operation cycles. Seismicity is detected using the matched filter technique (MFT) and located using waveform-correlation-based double-difference methods. This study extends previous research conducted by Tang et al*.*^16^ and Zhou et al*.*^22^ by investigating seismic activity over a longer period, providing insight into the evolution of seismicity over multiple UGS cycles, and helping to facilitate safer UGS operation.

## Geological settings and seismic observation

### Geologic settings

**Figure 1:**
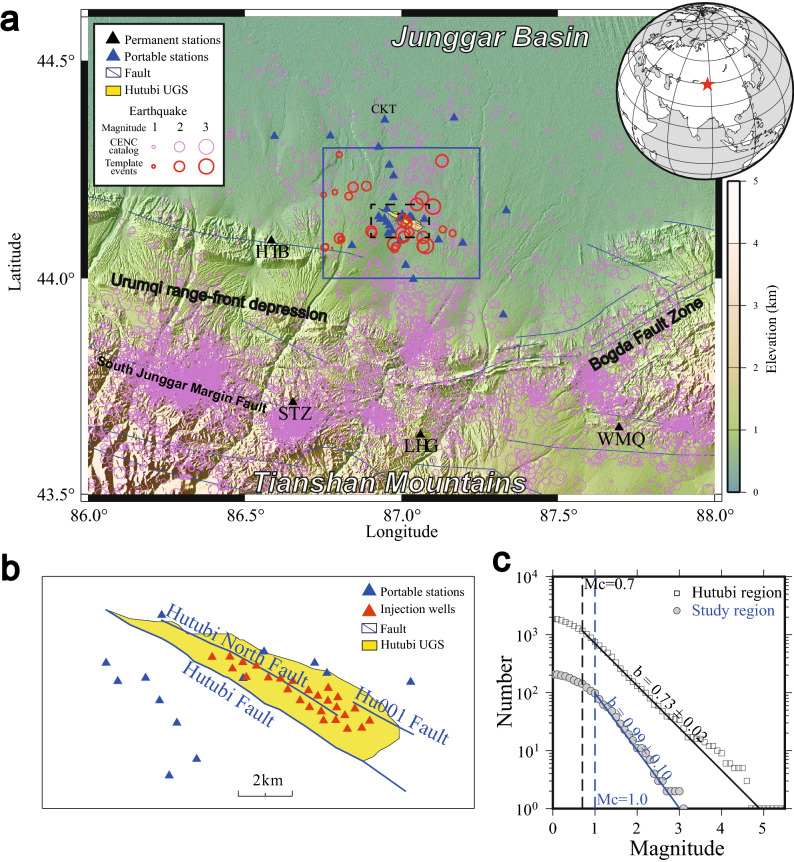
(**a**) Map of the study region. Portable and permanent seismic stations are marked with blue and black triangles, respectively. The yellow area is the surface projection of the Hutubi underground gas storage (UGS) facility. The blue rectangle encompasses the study area. The pink and red cycles show the earthquakes reported by the China Earthquake Networks Center and the relocated template events, respectively. The blue lines show the major faults in the region. (**b**) Detailed representation of the area around the Hutubi UGS, corresponding to the dashed rectangle in (**a**). The blue and red triangles are portable seismic stations and injection wells, respectively. (**c**) The cumulative number of earthquakes versus magnitude is fit to the Gutenberg-Richter relation to estimate the *M*_C_ and the b-value. (The elevation data is the 90 m topography from the Shuttle Radar Topography Mission^31^, this figure was created with the Generic Mapping Tools (GMT) 5.4.4, URL: www.generic-mapping-tools.org).

As the first gas storage facility along the second pipeline of the West–East Gas Transmission Project^21,23^, the Hutubi UGS is located on the southern edge of the Junggar Basin, a large superimposed basin adjacent to the northern Tianshan Mountains in western China^24^ (Fig. 1a). Created by the collision of the Indian Ocean and Eurasian plates in the Cenozoic era, the Tianshan Mountains have experienced strong compression and uplift and formed an active intracontinental regenerative orogenic belt^25–28^. To the northern edge of the Tianshan Mountains are located the southern Junggar Margin Fault and the Urumqi range-front depression (Fig. 1a). Within this depression are three groups of thrusting fault-anticline tectonic belts, which are separated by synclines^29,30^.

The Hutubi UGS is situated on top of the east–west extending Hutubi anticline, which is the latest active anticline in the northeast corner of the Urumqi range-front depression (Fig. 1a). The Hutubi anticline has a 40 km long axis, an 8 km short axis, and wing dipping of 6–15 degrees^29^. The reservoir formation of the Hutubi UGS is the Ziniquanzi formation, which is ~ 3585 m deep^21,23^. The Ziniquanzi formation is more than 300 m thick^23^ and is mainly composed of fine sandstone and inequigranular sandstone with a porosity of 5.3–22.4%^32^.

The Hutubi region is characterized by prevailing east–west striking and south-dipping reverse faults^30^. Three parallel reverse faults (Hutubi Fault, Hutubi North Fault, and Hu001 Fault), extending along the axis of the Hutubi anticline, cut through the Ziniquanzi formation (Fig. 1b). The Hutubi Fault is the major fault in this area, which forms the southern boundary of the reservoir (Fig. 1b). It is a ~ 35-degree south-dipping reverse fault extending approximately 20 km east–west^23,33^. The maximum fault displacement of the Hutubi Fault is close to 200 m^23,33^, while the Hutubi North Fault and Hu001 Fault have shorter lengths and smaller displacements^33^.

### Construction of the Hutubi UGS

The Hutubi UGS was constructed at the depleted Hutubi gas field discovered in 1996. Between November 1998 and April 2012, the Hutubi gas field produced approximately 5.9 billion m^3^ of natural gas and 220 thousand tons of crude oil with a recovery rate of ~ 48%^23^. The pore pressure in the reservoir formation decreased from ~ 34 to ~ 17 MPa following the production of oil and gas.

To bridge the gap between natural gas consumption and supply, the Hutubi gas field was converted into a UGS once the production had started to decline^21^. The total storage and throughput capacity of the Hutubi UGS are 10.7 billion m^3^ and 4.51 billion m^3^, respectively^21,23^. The construction included the capping of 10 existing wells and the drilling of 37 new wells. The drilling of the new wells began in June 2011, and well capping was completed on May 16, 2013^21^.

**Figure 2:**
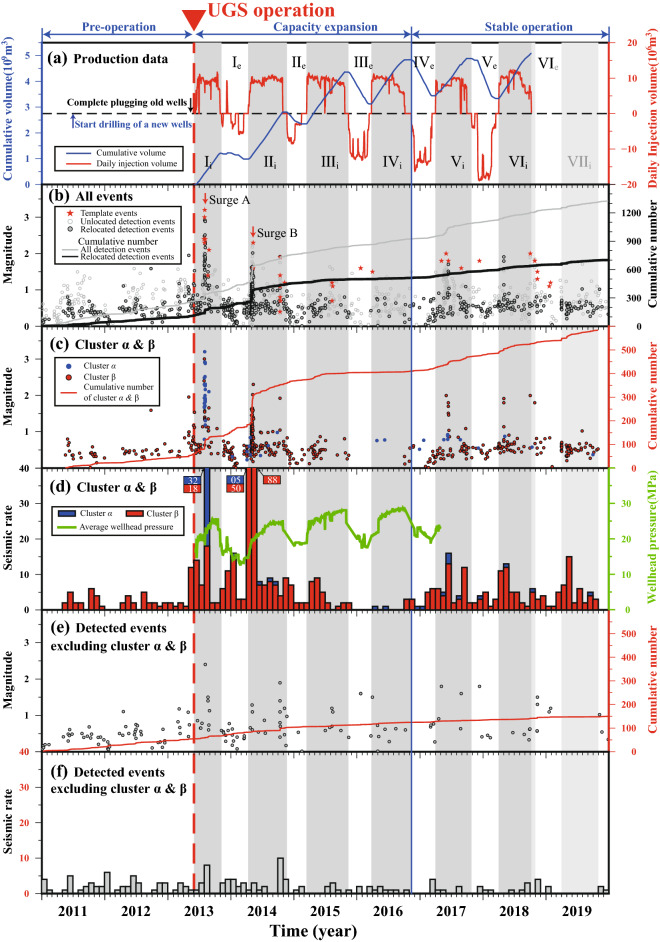
(**a**) The injection volume of the UGS during the study period. The red dashed line indicates the start of the Hutubi UGS operation (June 2013). The operation was divided into five complete cycles and half of a sixth cycle (I–V and VI_i_) spanning the capacity expansion and stable operation stages. Each cycle includes two periods: the injection period (with subscript i, gray area) and extraction period (with subscript e, white area). (**b**) An M–t diagram of detected (gray dots) and relocated catalog (black dots) events. The cumulative number of the detected and relocated catalogs are represented by the black and gray lines, respectively. The red stars indicate the template events. An M–t diagram (**c**) and monthly seismic rates (**d**) for two clusters. The cumulative number and the average wellhead pressure are represented by the red (**c**) and green (**d**) lines, respectively. The M–t diagram (**e**) and monthly seismic rate (**f**) for the seismic events excluding the two clusters. The cumulative number is represented by the red curve.

The Hutubi UGS became operational in June 2013. According to the production data (illustrated by the injection rate in Fig. 2a), the operation of the Hutubi UGS from June 2013 to October 2018 can be divided into five complete operation cycles (I–V in Fig. 2a) and one injection period (VI_i_ in Fig. 2a). Each complete operation cycle includes injection from April to October and extraction from November to the following March. Data on production for the period following October 2018 are hard to obtain. We estimated the sixth complete injection-extraction cycle and the seventh injection period by extrapolating past operation patterns. The UGS experienced two stages: the capacity expansion stage (from June 2013 to October 2016, including cycles I–III and injection period IV_i_) with net capacity (the blue curve in Fig. 2a) gradually increasing to ~ 5 billion m^3^, and the stable operation stage (from November 2016) with net capacity maintained at ~ 5 billion m^3^ (Fig. 2a).

### Seismic observation

In June 2013, we deployed 10 portable seismic stations to better monitor seismicity around the Hutubi UGS. By the end of 2019, 30 stations were in operation (Fig. 1a). Different seismometers were used according to instrument availability (Fig. S1). Unfortunately, the stations were poorly maintained in the early stages, and there were serious data gaps (Fig. S1). To fill these gaps, we also included four permanent broadband stations (illustrated by the black triangles in Fig. 1a) within 100 km from the UGS and one portable station (CKT in Fig. 1a) used for the airgun source signal recording^34^. The CKT station, which has not been used in previous studies^16,22^, remarkably improved the azimuthal coverage. All stations are 3-component with 100 samples per second. And all stations have flat response frequency covering the whole frequency band (2–8 Hz) used in our study.

## Earthquake detection and relocation

To investigate the seismicity around the Hutbubi UGS, we first detected the possible missing events using MFT, a waveform cross-correlation-based event detection method^35,36^. And then we relocated the detected events with double different relocation^37,38^.

### Earthquake detection

Although seismicity related to underground gas operation is seldom reported, seismicity induced by CO_2_ capture has been intensively studied^9^. Most studies suggest that CO_2_ injection^10^ and UGS-induced^13^ earthquakes usually occur within 10 km of the injection points. Therefore, we focused on an area within 10 km of the Hutubi UGS (illustrated by the blue rectangle in Fig. 1a). We obtained the local catalog from the China Earthquake Networks Center (CENC) within a larger area (43.5–44.6°N and 86.0–88.0°E) to investigate the background seismicity. From January 1, 2011, to December 31, 2019, the catalog recorded more than 5000 earthquakes (Fig. 1a), and 205 of these events were located in our study area. To determine the magnitude of completeness, we fitted the CENC catalog (for the whole area in Fig. 1a) with the Gutenberg–Richter relation using the ZMAP package^39,40^. The resultant magnitudes of completeness (*M*_C_) and b-value of the CENC catalog were 0.7 and 0.73 (Fig. 1c), respectively. The b-value is consistent with the b-value of the whole North Tianshan^41^; hereafter, we refer to this value as the background b-value.

The CENC catalog is obtained based on manual phase picking from the sparsely distributed permanent stations many kilometers away from the UGS^16^. Therefore, the event locations are poorly constrained, and many events may be omitted. To refine the catalog, we first located all the catalog events using the Hypoinverse program^42^ and further estimated the relative locations with the double-difference earthquake location technique HypoDD^37,38^ using data from permanent and portable stations (S2). Absolute locating was conducted using manually picked P- and S-wave arrival times. For the relative locating, we applied the waveform cross-correlation technique to the differential travel times between the event pairs^38^. In total, 330 events recorded were relocated, among which 34 events were located within our study area (Fig. 1a). We then re-evaluated the earthquake magnitudes according to the updated locations (S3). All 34 events were clearly recorded with a high signal-to-noise ratio (SNR > 3) on more than nine channels at permanent stations.

In the MFT detection, we used continuous data from four permanent stations and the relocated 34 events as templates (Fig. 1). The continuous and template waveforms were first band-pass filtered from 2 to 8 Hz. Then, the sliding window cross-correlations (CCs) between the templates and continuous waveforms were calculated at each channel. The time windows for the CC calculation were set to 1 s before and 3 s after the P- and S-wave arrivals for the vertical and two horizontal channels, respectively. Next, the continuous CCs were shifted according to the phase travel times of the template event and then averaged. Waveforms with an average CC value greater than 0.3 and 11 times greater than the median absolute deviation^36^ were regarded as a positive detection. To minimize the possibility of duplicate detections, we only kept the detection with the maximum CC in each 2-s time window^36^. Detected waveforms were then manually checked (Fig. S5), and 64 candidates were confirmed as false detections and discarded. The magnitude of the detected events was determined based on the median value of the peak amplitude ratios between the detected event and the template event for all channels^36^.

In total, we detected 1325 events from January 1, 2011 to December 31, 2019, more than six times the 205 events recorded in the CENC catalog for the same area and time period. All the catalog events were successfully detected (Fig. S6). The corresponding *M*_C_ and b-value of the detected catalog are 0.6 and 1.06 (Fig. S6), respectively. The b-value of the study area is higher than the background value (0.73) (Fig. 1c).

### Relocating detected earthquakes

We further relocated the detected events using HypoDD^37,38^ with fine-tuned local P- and S-wave velocity structures^34^. The initial location of each detected event was assigned to the location of the corresponding template. The differential travel times of all events pairs at each station (permanent or portable) were measured through waveform (2–8 Hz filtered) cross-correlation^38^ with 2-s (0.5 s before and 1.5 s after) and 3-s (1 s before and 2 s after) time windows for P- and S-waves, respectively. Differential travel times with CC > 0.4 were used for the relative location, and it imposes strong constraints on the relative event locations, which significantly improves the location accuracy.

In total, 790 of 1325 detected events were relocated, and 684 of them were located within the study area. This relocation process reduced the travel-time residual from 2.51 to 0.22 s. The average horizontal and vertical location precision were estimated^37^ as 0.27 km and 0.30 km, respectively (Fig. S7).

## Spatiotemporal evolution of seismicity around the Hutubi UGS

### Temporal evolution

More than half of the detected events were relocated inside our study area and the relocated events show a similar temporal pattern to the entire detected catalog (Fig. 2b). In the beginning, the seismic rate was low (less than 10 events per month) but abruptly increased after the UGS operation began (Fig. 2).

During the capacity expansion stage (June 2013–November 2016), the seismicity was dominated by two distinct surges (A and B in Fig. 2b). The first seismic surge (A in Fig. 2b) occurred two months after the beginning of the first gas injection (Fig. 2d), and the seismic sequence lasted for ~ 10 days. The second seismic surge (B in Fig. 2b) occurred about 20 days after the second gas injection and lasted for ~ 20 days. Both seismic surges were associated with sharp wellhead pressure increases (Fig. 2d). After the two surges, the seismicity gradually weakened (Fig. 2b), with only a few events occurring after the injection period of cycle III (Fig. 2).

When the net capacity reached its maximum at the end of the fourth injection period, the UGS moved into its stable stage, and the seismicity subsequently recovered (Fig. 2). The events in the stable stage exhibited low magnitudes (*M*_L_ < 2.0) and were mainly associated with gas injection rate changes (Fig. 2).

### Hypocenter distribution

**Figure 3:**
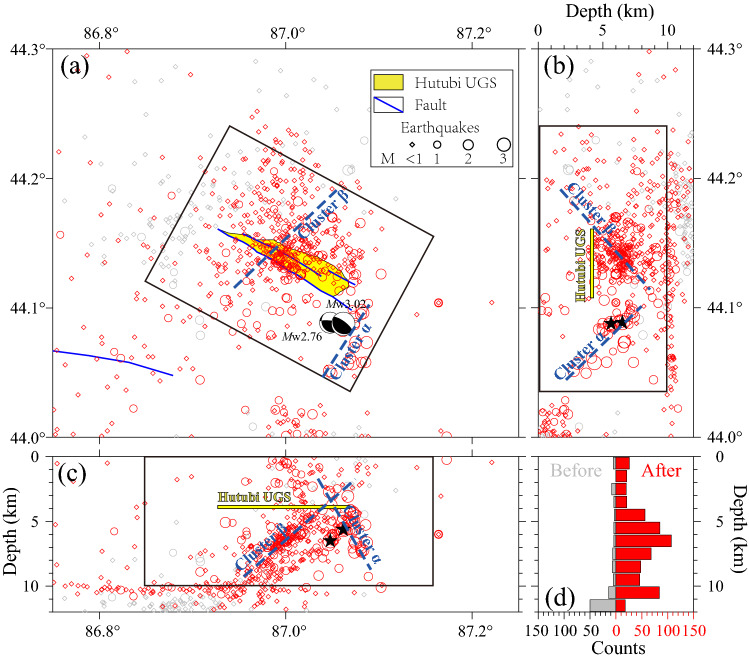
The map view of the relocated events. (**a**–**c**) The distribution of all relocated events. The events where *M*_L_ > 1.0 are marked by magnitude-scaled circles, and smaller events (*M*_L_ < 1.0) are represented by diamonds. The two beach balls in (**a**) represent the focal mechanisms of largest events (*M*_L_3.4 and *M*_L_3.1 in August 2013) from Zhou et al*.*^22^. The yellow area marks the Hutubi UGS. (**d**) The depth distribution of events before and after the start of the UGS operation is shown by the gray and red histograms, respectively.

The earthquakes in the study area are mainly distributed at a depth of 4–12 km, with two peaks at 7 km and 11 km (Fig. 3d). Horizontally, the shallow events (< 10 km deep) are widely distributed within 6 km of the UGS (the black rectangle in Fig. 3). These were rarely observed before the start of the UGS operation (Fig. 3d). Deep events (> 10 km deep) are mainly located west of the UGS and are consistently active throughout the study period with a low magnitude (*M*_L_ < 1.0) (Fig. 2e) and low seismic rate (less than 10 events per month) (Fig. 2f).

**Figure 4:**
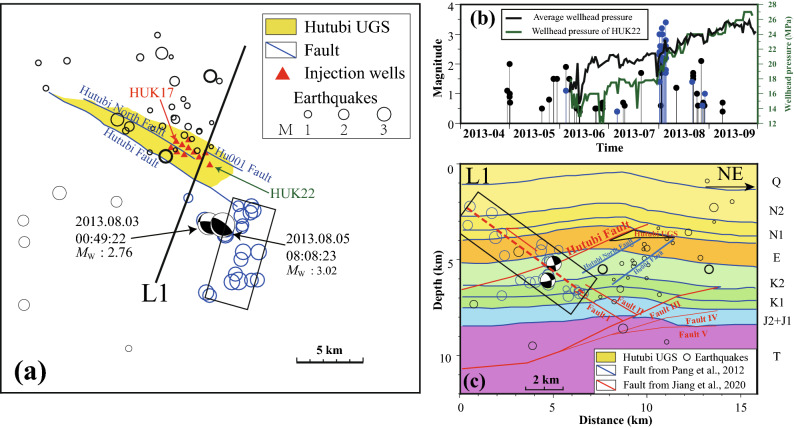
(**a**) The map view, (**b**) M–t diagram, and (**c**) profile along L1 in (**a**) of seismicity during the first injection period. Blue and black cycles are events belonging to clusters α and β, respectively. The average wellhead pressure and the wellhead pressure of HUK22 in (**a**) are shown in (**b**) as black curve and dark green curve, respectively. Beach balls in (**a**) and (**c**) correspond to the focal mechanisms of the two largest events from Zhou et al*.*^22^. The black box indicated the seismic zone formed by the cluster α. The solid red and blue lines in (**c**) are faults interpreted from the seismic reflection profile described by Jiang et al*.*^18^ and faults inferred from seismic and geologic surveys^23,33^, respectively. The dashed red line depicts an extension of cluster α.

The events within 6 km of the UGS can be further divided into two clusters (α and β in Fig. 2). The aforementioned surge A is a subset of cluster α, which hosted more than 60% of the events in this cluster (Fig. 2d). Cluster α is concentrated along a ~ 5 km, ~ 30-degrees northeast-dipping plane, with the plane conjugate to the Hutubi Fault (Figs. 3 and 4c). Cluster β mainly occurred beneath the UGS reservoir along a southwest-dipping plane with a ~ 35-degree angle, with the plane parallel to the Hutubi North Fault (Figs. 7 , S8). Cluster β was active throughout the study period and hosted most events during the stable operation stage (Figs. 2c and S8). The events in surge B mainly occurred in cluster β (Fig. 2c). The P-wave cross-correlation coefficients between intra-cluster events were higher than between inter-cluster events (see S8), which indicated that the seismogenic structures of intra-cluster events were more similar than inter-cluster events.

## Discussion

### Overall seismicity evolution and Hutubi UGS operation

Although located in the Tianshan Mountains seismic zone^25,27^, the study area was seismically quiet before the start of the Hutubi UGS operation, with a monthly seismic rate of less than five (Fig. 2; Tang et al*.*^16^). However, seismicity significantly increased after the UGS operation began (Fig. 2). In particular, two seismic surges (A and B) during the capacity expansion stage experienced more than 50 events per month, and the seismicity during the UGS operation showed a strong correlation with gas injection (Fig. 2).

To investigate the statistical features of seismicity, we modeled the relocated catalog with the Epidemic-Type Aftershock Sequence (ETAS)^43,44^. The ETAS model (see S9) shows a high total forcing rate (~ 79%), indicating that the majority of seismic events were externally triggered^6,45,46^. This is similar to the high forcing rate observed for seismic events induced by fluid (wastewater) injection^6,45^.

Both tectonic loading and injection stress account for these external forces. Tectonic loading is almost constant, while injection is typically intermittent^6^. To address this temporal variation, we further adopted the ETAS model with a time-varying forcing rate^6,46^. The forcing rate obtained shows a good correlation with the gas injection process (Fig. S12). Increased seismicity and a strong coherence between the force rate and the injection period indicate the relationship between seismicity and UGS operation.

The two clusters (α and β, illustrated by the black rectangles in Fig. 3) mainly occurred at a shallow depth of < 10 km and in close proximity (< 6 km) to the boundary of the Hutubi UGS reservoir. Compared to the seismicity in clusters α and β, the seismic rate further (> 6 km) from the UGS was stable and low, with a monthly rate of less than 10 for the whole study period (Fig. 2f).

These observations suggest that UGS operation modified seismicity within ~ 6 km of the UGS. This distance is similar to that reported for the Castor UGS^13^ and for CO_2_ storage facilities^10^ but smaller than the range determined for fluid injection^1,47^.

By including the airgun station (CKT, Fig. 1), we could obtain better azimuthal coverage and more reliable event locations (Fig. S14). Compared to previous studies^16,22^, our locations result in lower travel-time residuals (Fig. S14). The two event clusters (Fig. 3) demonstrate different spatiotemporal patterns (Figs. 2d, 3) may be attributed to different seismogenic structures.

### The initiation of two clusters

Cluster α initiated with the rapid wellhead pressure increase (Fig. 4b) in the HUK22 (the easternmost injection well during the first injection period, Fig. 4a), which represents the pressure change of all wells (Fig. 4b). This cluster hosted the two largest events within the area and time period of the study, which attracted much concern^16^. According to the focal mechanisms, Zhou et al*.*^22^ argued that these two events were sliding along an unmapped south-dipping reverse fault parallel to the Hutubi Fault. While our relocation indicates that the hypocenter in cluster α formed a narrow (~ 3 km wide) north-dipping seismic zone southeast of the UGS at depth of 2–7 km (Fig. 4). This seismic plane is conjugate to the Hutubi Fault (~ 30-degree dipping) and is consistent with the extension of two minor faults (I and II in Fig. 4c) mapped by the seismic survey. Therefore, we argue that the cluster α (box in Fig. 4c) occurred along the existing north-dipping reverse fault.

Cluster β hosted more than 90% of the events surrounding the UGS (Fig. 2d). Cluster β demonstrates a southwest-dipping plane beneath the UGS reservoir (Figs. S7 and S8) at depth of 4–8 km. The plane has a dipping angle of ~ 35 degrees and is consistent with a westerly extension of the Hu001 Fault (Figs. 7, S8). The seismicity of the cluster β is visible during the whole study period and was enhanced since the start of the UGS operation (Fig. 2d). The seismic rates in cluster β exhibit a strong correlation with average wellhead pressure, with more than 77% of events occurring during the injection period (Fig. 2d).

**Figure 5:**
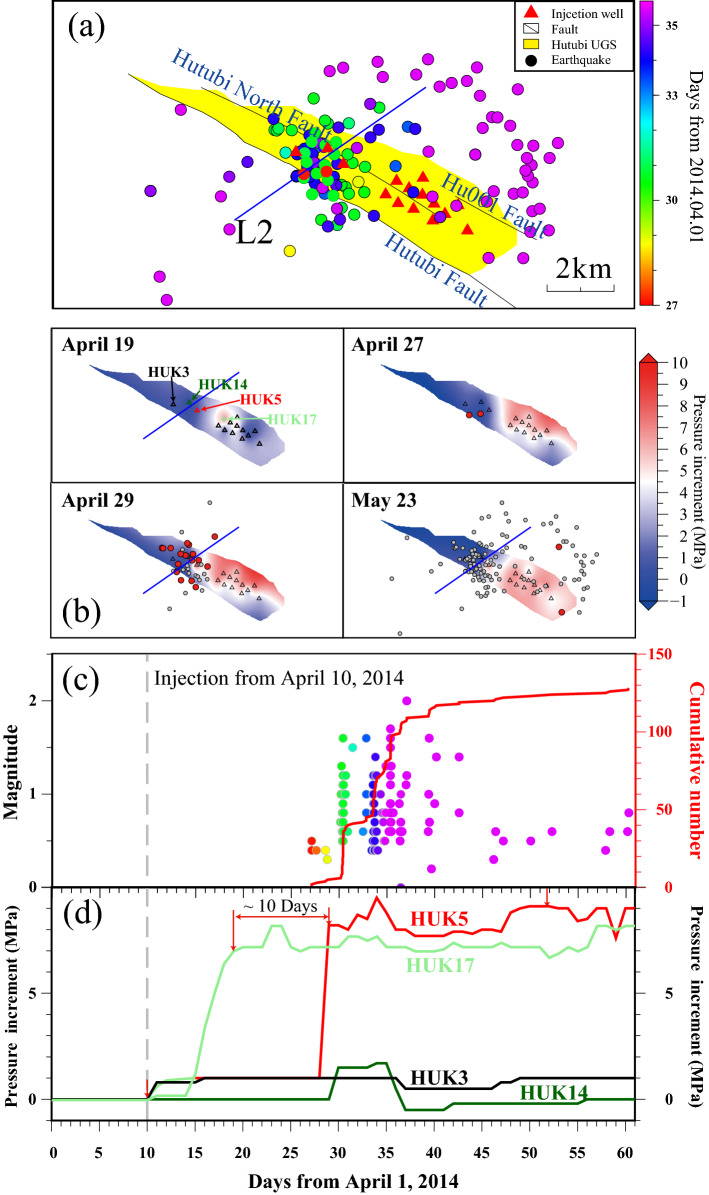
The map view (**a**) and M–t diagram (**c**) of the surge B seismic events occurring between April 1, 2014 and May 31, 2014. The injection wells operated during the second cycle are marked as red triangles in (**a**). The blue line L2 in (**a**) and (**b**) marks the position of profile shown in Fig. 7b and c. (**b**) The reservoir gas pressure distribution interpolated from the wellhead pressure increments relative to April 6, 2014. Current and previous earthquakes are marked as red and grey cycles, respectively. Triangles are injection wells in operation during the second injection period. (**d**) The pressure increments of the four representative wells. The gas injection started from the HUK17, and other wells began gas injection successively in the following 10 days. Three wells (HUK5, HUK3, and HUK14) were closely located in the western part of the reservoir but had prominent different well pressure.

The seismicity of cluster β occurred close to HUK17 gas injection well during the first gas injection period (Fig. 4a), while the earthquake sequences in cluster β during the second injection period was initiated close to HUK5 (Fig. 5). The well HUK5 is about 1.6 km northwest to HUK17 (Fig. 5b) and was not operated prior to the second gas injection period (Fig. 4a). Both two seismicity bursts in cluster β were initiated approximately 3 km beneath the UGS reservoir (black circles in Figs. 4c and S8).

**Figure 6:**
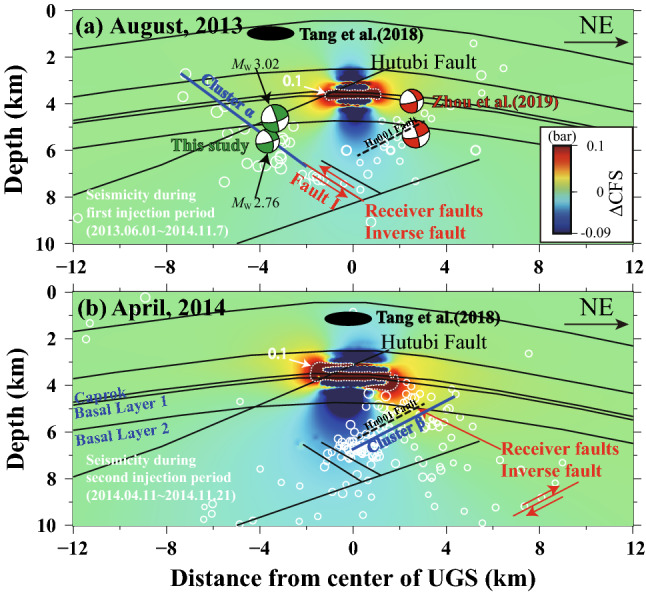
Accumulative Coulomb stress perturbations caused by the operation of the Hutubi UGS were estimated with a hydrogeomechanical model^18^ for clusters α (**a**) and β (**b**). The putative receiver faults are set as northeast-dipping with 30° inverse fault and southwest-dipping with 20° inverse fault, and the friction coefficient was fixed at 0.4. White dashed lines mark the boundaries of 0.1 bar increments. The black ellipse shows the location of two clusters reported by Tang et al*.*^16^. The beach balls represent focal mechanisms of two earthquakes with *M*_L_ ≥ 3.0 in August 2013, plotted in the locations described by Zhou et al*.*^22^ (red) and by this study (green). The white circles indicate the events during the first (**a**) and second (**b**) injection periods. The two planes outlined by the two clusters are shown as blue lines for the different operating phases. The black dashed lines mark the Hu001 fault, although it is not considered in the hydrogeomechanical model^18^.

Increasing seismicity has been attributed to pore pressure changes^16^ or poroelastic stress perturbations^22^. Our results show that cluster α is more than 2 km from the UGS reservoir (Fig. 4c), and the initiation point of the cluster β is about 3 km deeper than the reservoir (Fig. 6b). There is no clear evidence of a fluid connection between the reservoir and the two clusters. Therefore, pore pressure diffusion-induced fault weakening is unlikely the main mechanism behind the occurrence of the seismicity.

The injection process results in instant elastic stress changes and delayed poroelastic stress perturbations, they can alter the Coulomb failure stress (△CFS) on potential faults and bring the faults to failure^1^. We evaluated the injection induced accumulated Coulomb failure stress changes on the two clusters (Fig. 6) with a two-dimensional hydrogeomechanical model developed by Jiang et al.^18^. The model indicated that the cluster α was initiated from the area with a weak positive △CFS (about 0.7 kPa) (Fig. 6a), the increase is much smaller than the widely accepted minimum failure stress increment (10 kPa). And cluster β initiated from a negative △CFS area (Fig. 6b). Negative △CFS suggests lower earthquake risk in general^18^.

Though the two clusters are not initiated from areas with high positive △CFS, the two clusters showed temporal correlations with the wellhead pressure changes (Figs. 4b and 5) and they were initiated close the gas injection boundaries. Before and during the second injection period, the wellhead pressure of wells (e.g., HUK22, HUK17, and HUK5 that was in the first operation during the second injection period) in the eastern part were raising with the natural gas injection, while wells in the western part (i.e., HUK14 and HUK3) did not show any observable pressure change (Fig. 5). Cluster α was located ~ 3 km from the east boundary UGS region, where there is no operation or monitoring well (Fig. 4). Two earthquake sequences in cluster β during the first two injection periods were initiated from the westernmost injection well (Figs. 4a and 5a). Bounded gas injections are expected to generate strong pressure gradients and strain localization along the injection boundaries (Fig. 5b and Videos S1), which was not consider in the 2-D hydrogeomechanical model. Both boundaries extended SW, in accordance with the extensions of the two clusters’ initiations. Strain localizations are believed to relate to small earthquakes^48,49^. Although we still lack enough information to carry out detailed 3-D modeling, we propose that the elastic and poroelastic stress changes together with injection induced strain localizations are responsible for the initiation of the two clusters during the capacity expansion stage.

### The migration of cluster β during the second injection period

**Figure 7:**
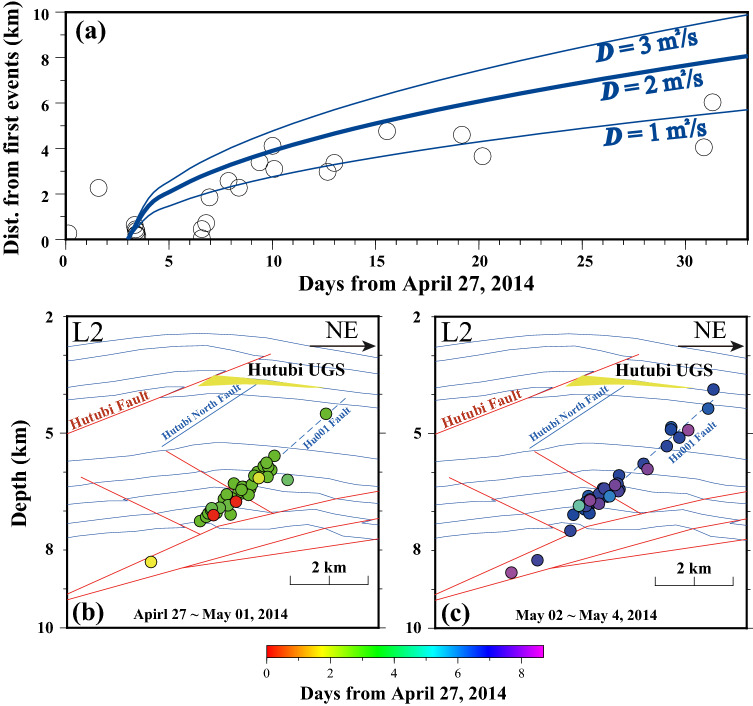
(**a**) The relationship between origin time and distance from the first event in cluster β during the second injection period. The blue lines correspond to diffusion models (Eq. 1) with different hydraulic diffusivity *D*^50^, and the best fit hydraulic diffusivity *(D* = 2 m^2^/s) is shown by the bold curve. The blue lines correspond to diffusion models (Eq. 1) with different hydraulic diffusivity *D*^50^. (**b**) and (**c**) The event distributions at different time periods along L2 (Fig. 5a). Earthquakes are denoted by solid circles and colored by their origin time related to April 27, 2014.

Since cluster α faded out soon, cluster β dominates the seismicity in our study area. Events in cluster β gradually migrated to the northeast of the UGS during the second injection period (Fig. 5a and Videos S1), this migration was not mentioned in previous studies. Within ~ 10 days (Fig. 7a), the events migrated ~ 4 km to shallower parts along the western elongation of the Hu001 Fault (Fig. 7c). The △CFS in the shallow part is larger than in the deeper parts (Fig. 6b), which favorites the upward migration.

This type of seismic migration is generally attributed to fluid migration along faults^1^. The pore pressure front diffusion in porous media can be simplified as:

1 $$r = \sqrt {4\pi Dt}$$

where *r*, *D*, and *t* are migrating distance, diffusivity, and migrating time, respectively^50^. We fit the migration front with Eq. (1) and estimated *D* = 2 m^2^/s (Fig. 7a), the diffusivity is located within the range of typical crustal diffusivity (0.01–10 m^2^/s)^51^. We then further estimated the corresponding permeability ~ 2 × 10 ^− 14^ m^2^ (see S11). The estimated permeability is three to four orders higher than the permeability for the basal layers (I and II in Fig. 4) but is in good agreement with the permeability of the fault zone, which ranges from 10 ^− 12^ to 10 ^− 15^ m^2^^18,52^. Therefore, it is reasonable to believe that the seismic sequence occurred along a preexisting fault.

**Figure 8:**
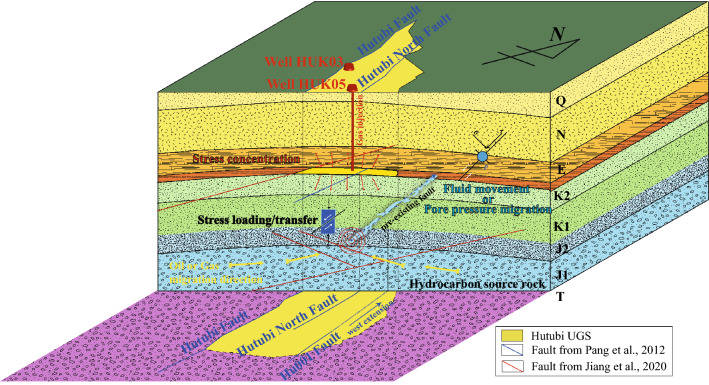
Schematic illustration of the hypocenter migration during the second injection period. The rocks in the hydrocarbon source formation are likely porous and may be partially saturated with fluids. Disturbed by the UGS loadings, microcracks begin to cohere along the preexisting fault, leading to fault sliding (earthquake). This sliding promotes increased pore-fluid migration along the existing fault, driving the earthquake migration to a shallower depth.

Since the earthquake sequence initiated from the Jurassic strata (Fig. 8), which is the hydrocarbon source formation of the Hutubi gas field, the formation is likely to be porous and partially saturated^53,54^. Disturbed by the injection, cracks in the formation may open and release fluids (Fig. 8). These fluids may leak into the preexisting fault and migrate from deeper to shallower parts driven by the confining pressure (Fig. 8). This will cause the redistribution of pore pressure and weaken the fault^55^ (Fig. 8).

### The seismicity of cluster β during the stable operation stage

Following the two seismic surges, seismicity around the UGS gradually weakened (Fig. 2d), and the seismic rate during the fourth injection period (< 10 events per month) was even lower than the seismic rate before the UGS operation (Fig. 2d).

When entering the stable operation stage, the net capacity reached ~ 5 billion m^3^, and the wellhead pressure peaked at ~ 30 MPa, which was close to the pore pressure observed prior to gas extraction^23^. Cluster β was reactivated during the stable operation stage (Fig. 2c) with enhanced seismicity with abrupt injection rate increases (Fig. 2d). The repeated injection and extraction of gas in the UGS may cause stress redistribution near the reservoir^19^, and lead to deformation and fracture propagation^56,57^. The deformation and fracture propagation processes are uneven, leaving the revived seismic events relatively scattered (Figs. S8 and S15).

It should be noted that both the rate and magnitude of the revived seismicity are lower than the aforementioned two previous seismic surges (Fig. 2c), and the seismic rate of earthquakes where *M*_L_ > 1.0 also decreased from seven per year to just one (Fig. 2c). Whether the stable net capacity will further weaken the operation-related seismicity is to be answered with longer-term observation.

## Conclusions

We detected and located the seismicity near the Hutubi gas storage from 2011 to 2019 using the portable and permanent seismic stations within 100 km. The seismicity remarkably increased after the UGS operation. And the effect of UGS operation is likely bounded within 6 km. The seismicity around the UGS can be grouped into two clusters and attributed to different seismogenic faults. Both clusters were initiated close to the boundaries of gas injection. The elastic and poroelastic stress changes together with the strain localization from non-uniform gas injection are likely responsible for the initiation of the seismicity. The seismicity during the second injection period showed clear migration to shallower parts, the migration is likely driven by the trapped crude oil/gas. After several cycles of operation, the seismicity tends to be stable and weak, occurring mainly along the major faults. Long-term monitoring is still needed to further investigate the UGS related seismicity, serving the safety in UGS production and local seismic hazard mitigation.

## Supplementary Information

Supplementary Information.

Supplementary Information.

## Data Availability

The data used in this study are collected by the Institute of Geophysics, China Earthquake Administration, which is available from the corresponding author (Email: bwgeo@ustc.edu.cn) upon reasonable request and with permission of the Institute of Geophysics, China Earthquake Administration.
