# Supplementary material for: Spatiotemporal evolution of seismicity during the cyclic operation of the Hutubi underground gas storage, Xinjiang, China
Source: Sci Rep. 2022 Aug 24;12:14427. doi: 10.1038/s41598-022-18508-x (PMC9402545; doi:10.1038/s41598-022-18508-x)
Supplement: Supplementary file 1 — Supplementary Information. [file 41598_2022_18508_MOESM1_ESM.pdf]

Supplementary Material for Spatiotemporal evolution of seismicity during the cyclic operation of the Hutubi underground gas storage, Xinjiang, China

Bo Zhang[1], Baoshan Wang[2,3,1*], Bin Wei[4], Zhide Wu[5], Ni-Er Wu[4], Renqi Lu[6], Zhanbo Ji[1,7], Jinxin Hou[1], Lu Li[1]

[1] Institute of Geophysics, China Earthquake Administration, Beijing, China.

[2] School of Earth and Space Sciences, University of Science and Technology of China, Hefei, China.

[3] Mengcheng National Geophysical Observatory, University of Science and Technology of China, Hefei, China.

[4] Earthquake Agency of Xinjiang Uygur Autonomous Region, Urumqi, China.

[5] Research Institute of Petroleum Exploration and Development, Langfang, China.

[6] Institute of Geology, China Earthquake Administration, Beijing, China

[7] Chinese Academy of Geological Sciences, Beijing, China

[*] Corresponding author: bwgeo@ustc.edu.cn

S1. The portable station recording

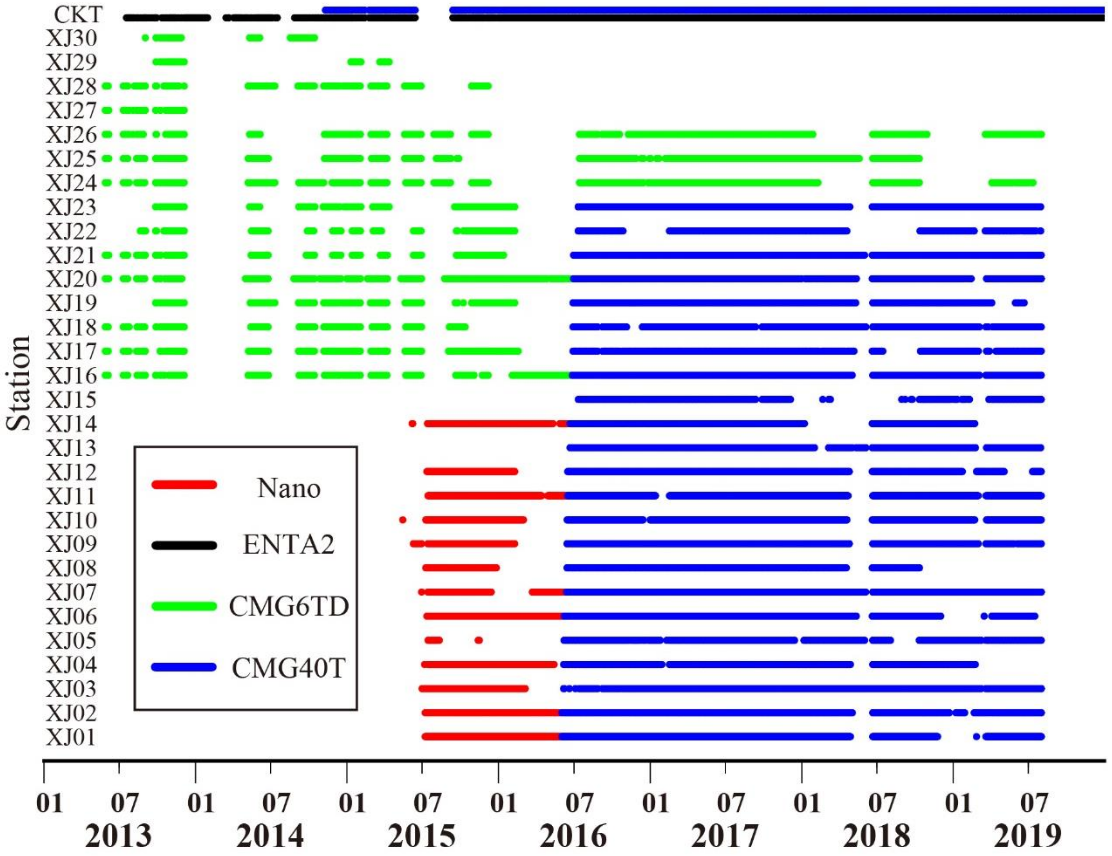

Figure S1. Data availability of portable seismic stations. Prior to June 2016, the stations were poorly maintained.

S2. Relocation of template events

We selected the catalog for a larger area than the study region (43.5–44.6°N and 86.5–87.5°W) considering the poor monitoring ability of the local permanent network[1,2]. Three hundred thirty earthquakes recorded by more than four stations (portable and permanent) were absolutely located using manually picked P- and S-wave. We located those earthquakes using the Hypoinverse algorithm[3] and the calibrated 1D velocity model[4]. The average standard deviations for the horizontal and vertical directions were 0.49 km and 0.52 km, respectively. The relocation results in location offset up to more than 20 km. One corresponding event reported at the north side of the Hutubi UGS by the local catalog was relocated to the south side of the Hutubi UGS (Figure S2) with offset 0.56 km. The new location fits the seismic profile better than the catalog location (Figure S2).

Based on absolute location, we further relocated all events using the double-difference relocation algorithm[5] to constrain relative locations. Differential P- and S-wave travel times between each earthquake were obtained using the waveform cross-correlation technique[6]. All waveform data were band-pass filtered from 2 to 8 Hz. The differential P-wave travel times were measured with 2-s long (0.5 s before and 1.5 s after the manual detection of the P-wave arrival time) and 1-s sliding windows. For the S-wave, the time windows were set to 1 s before and 2 s after the manual picks. In total, we obtained 111,425 P- and 122,976 S-wave differential times under the cross-correlation thresholds of 0.4 and 0.3, respectively.

Only 170 out of 330 events were located by the HypoDD algorithm, among which

34 events were located within our study area (Figure S3). The relocation process reduced the root mean square (RMS) of the travel-time residuals from 2.06 s to 0.43 s (Figure S4).

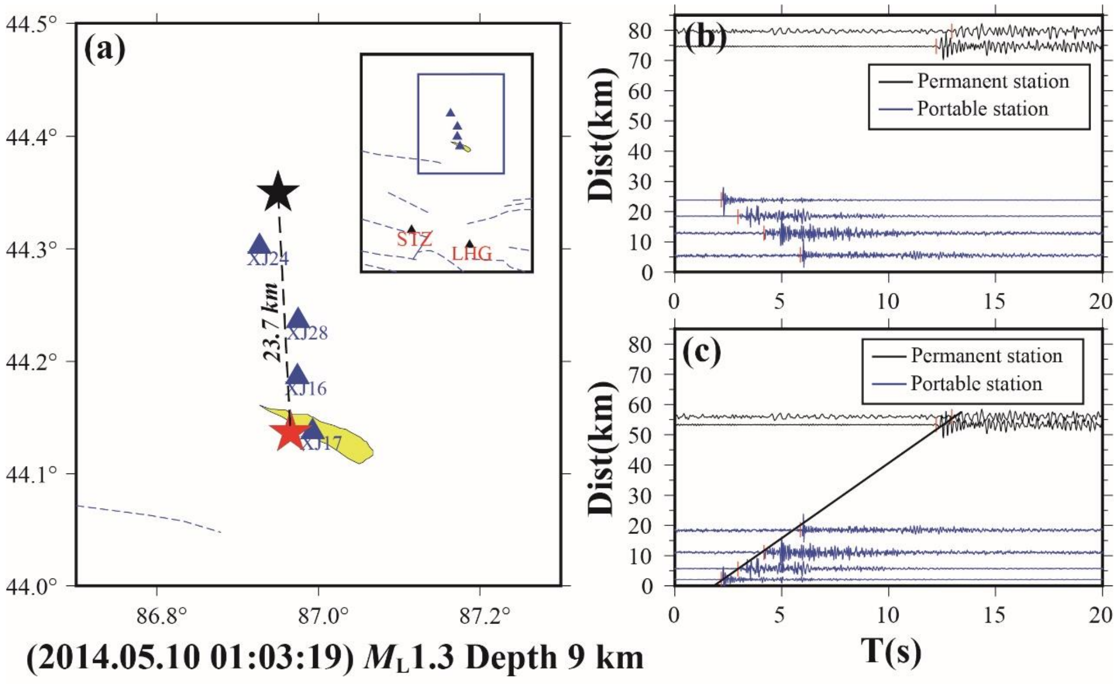

Figure S2. (a) The relocation result of the M_L1.3 event that occurred on May 10, 2014. The black star is the catalog location, and the red star is the relocated epicenter. The vertical component seismic profile is illustrated before **(b)** and after **(c)** the relocation. The blue and black waveforms are recorded by portable and permanent stations, respectively. P-wave arrival times picked manually for each station are marked by the red bars.

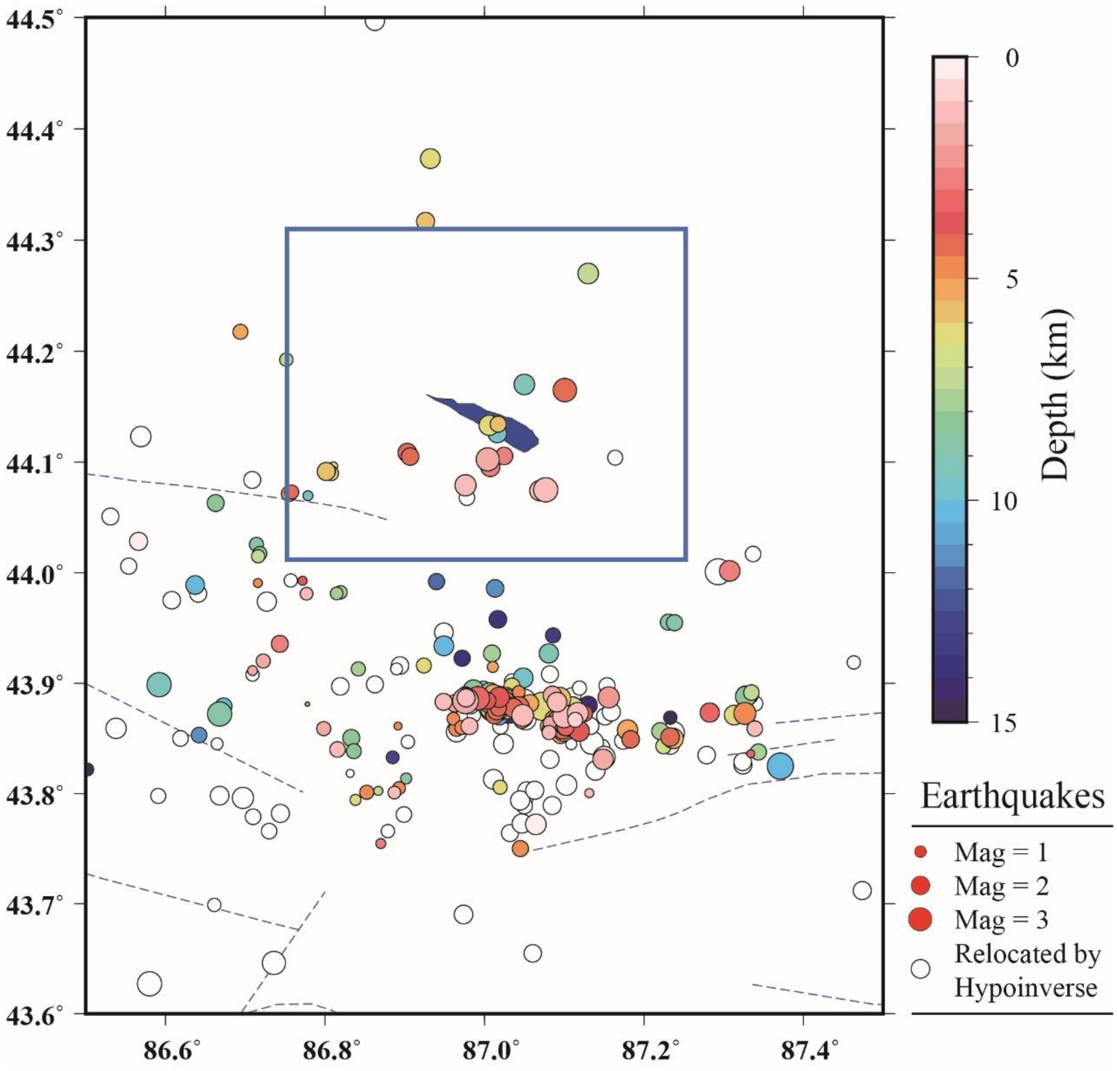

Figure S3. Relocation results of the catalog events. The events relocated by Hypoinverse and HypoDD are marked with hollow and solid cycles, respectively. The HypoDD-relocated events are scaled by their magnitude and colored according to their epicentral depth. The blue rectangle represents the study area.

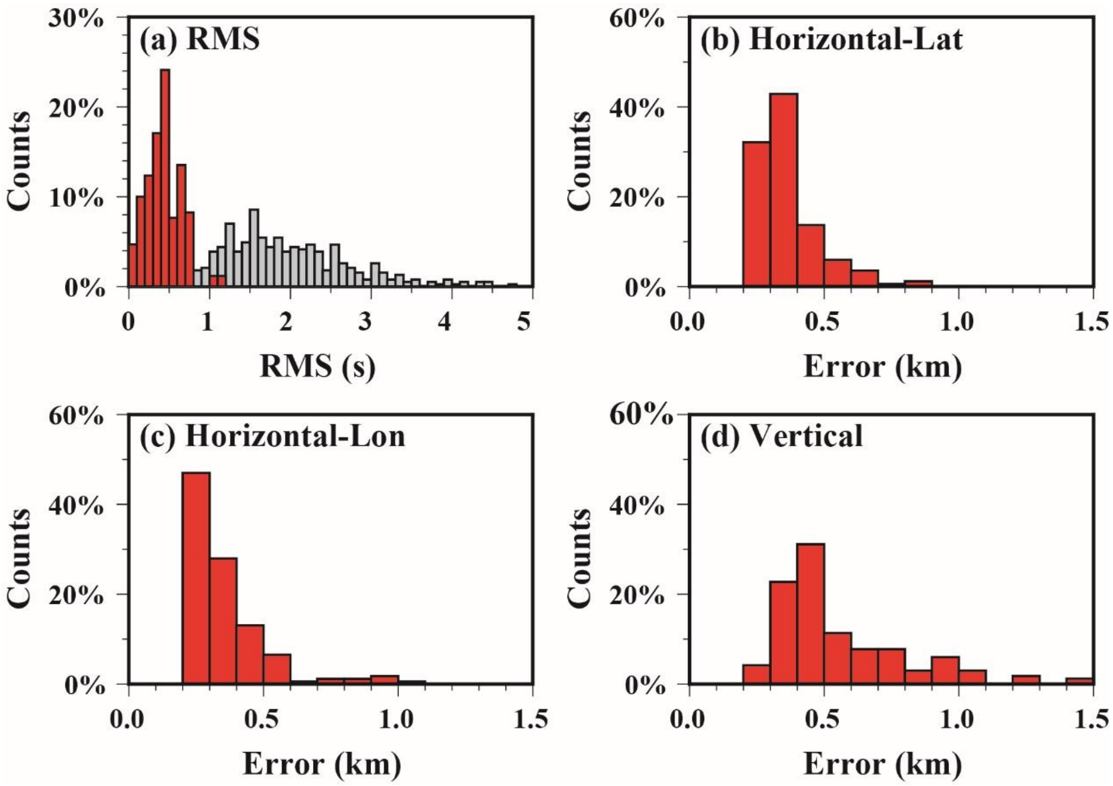

Figure S4. (a) Histograms of the root mean square (RMS) of travel-time residuals for the catalog (gray) and relocated (red) events. Location-error histograms in longitudinal **(b)**, latitudinal **(c)**, and vertical **(d)** directions.

S3. Re-estimating the magnitude

Since the relocation process offset the catalog events substantially, it was necessary to re-estimate the earthquake magnitudes. We used data from high-quality STZ and LHG stations (Figure 1) to calibrate the magnitude. Firstly, we convert the seismic record into the record of Wood-Anderson seismometers ($T_0 = 0.8$, V = 2,800, h = 0.8). The local magnitude could then be determined using the following equation:

$$M_L = lgA(\Delta) + R(\Delta), 30km \leq \Delta \leq 600km \ , \quad (1)$$

$$A = \frac{A_N + A_E}{2} \ , \quad (2)$$

where $A(\Delta)$ is the maximum average S-wave displacement of two horizontal (E and N) components, and $R(\Delta)$ is the gauge function proposed by Richter[7]. Using this technique, the magnitude of the event on May 10, 2014, was reduced from 1.5 to 1.3 (Figure S2).

S4. Example of detected events

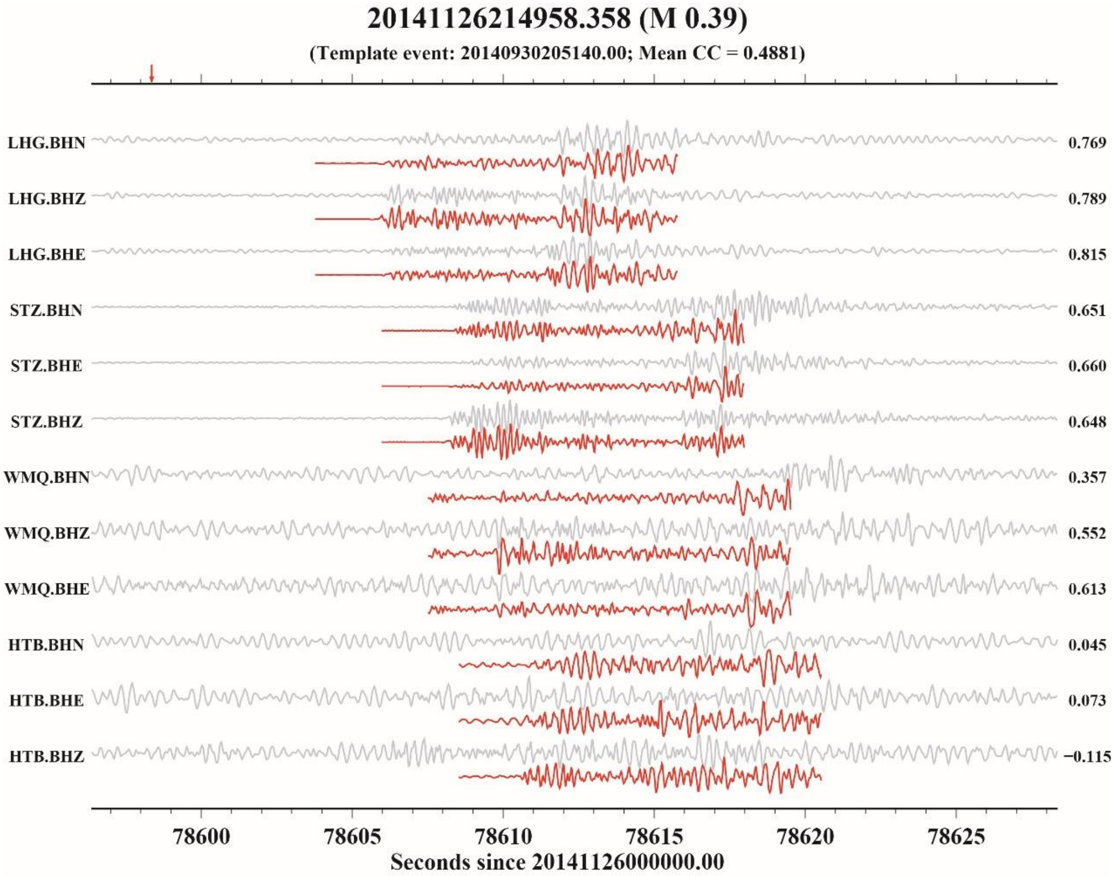

Figure S5. Example recording showing the detection of a M_L 0.39 earthquake with a M_L 2.1 template. The template traces (in red) are cross-correlated with the continuous traces (in black), the cross-correlation will reach a local maximum at a seismic event. The mean CC is marked at the right side of each waveform, and the origin time is labeled with a red arrow.

S5. The detected catalog and Gutenberg–Richter relation fitting

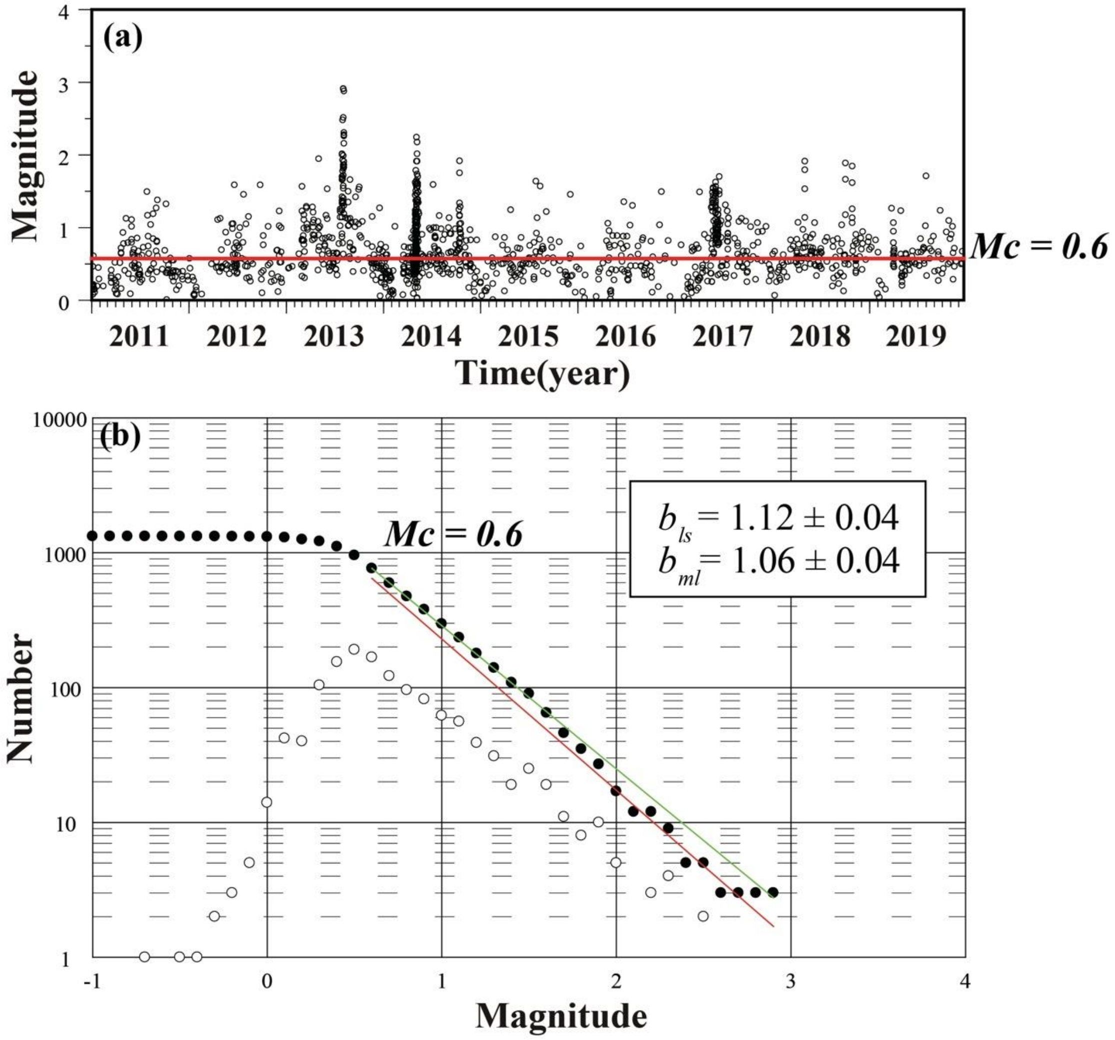

Figure S6. (a) The M–t diagram of the detected catalog where the red line indicates the magnitude of completeness (*M*c). **(b)** Frequency–magnitude distributions (open cycles) and Gutenberg–Richter relation fitting (solid cycles) of the detected catalog. The magnitude of completeness (*M*c) and b-value are calculated using the ZMAP package[8,9]. The b-values estimated using the maximum likelihood (b_{ml}, green line) and least-squares (b_{ls}, red line) methods are similar.

S6. Relocation error of detected events

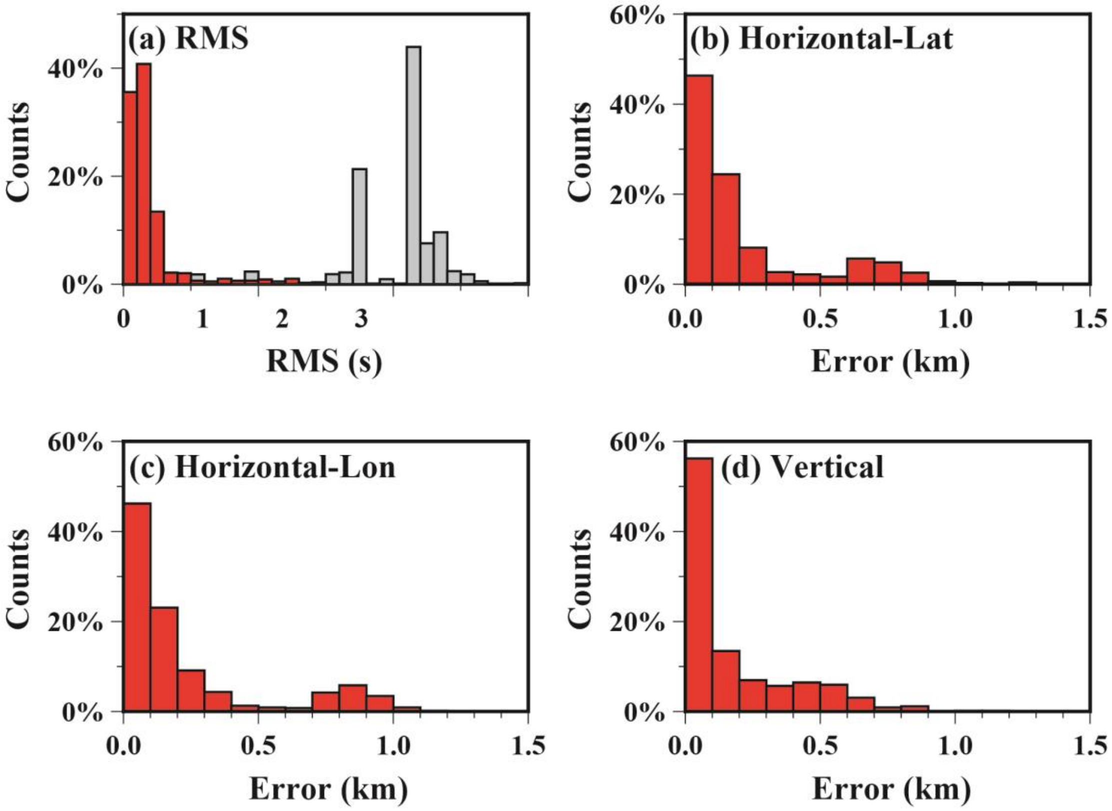

Figure S7. (a) Histograms of the root mean square (RMS) of travel-time residuals for the MFT detected catalog (grey) and relocated result (red). Location-error histograms in longitudinal **(b)**, latitudinal **(c)**, and vertical **(d)** directions.

S7. Section views along L2 of cluster β at different time periods.

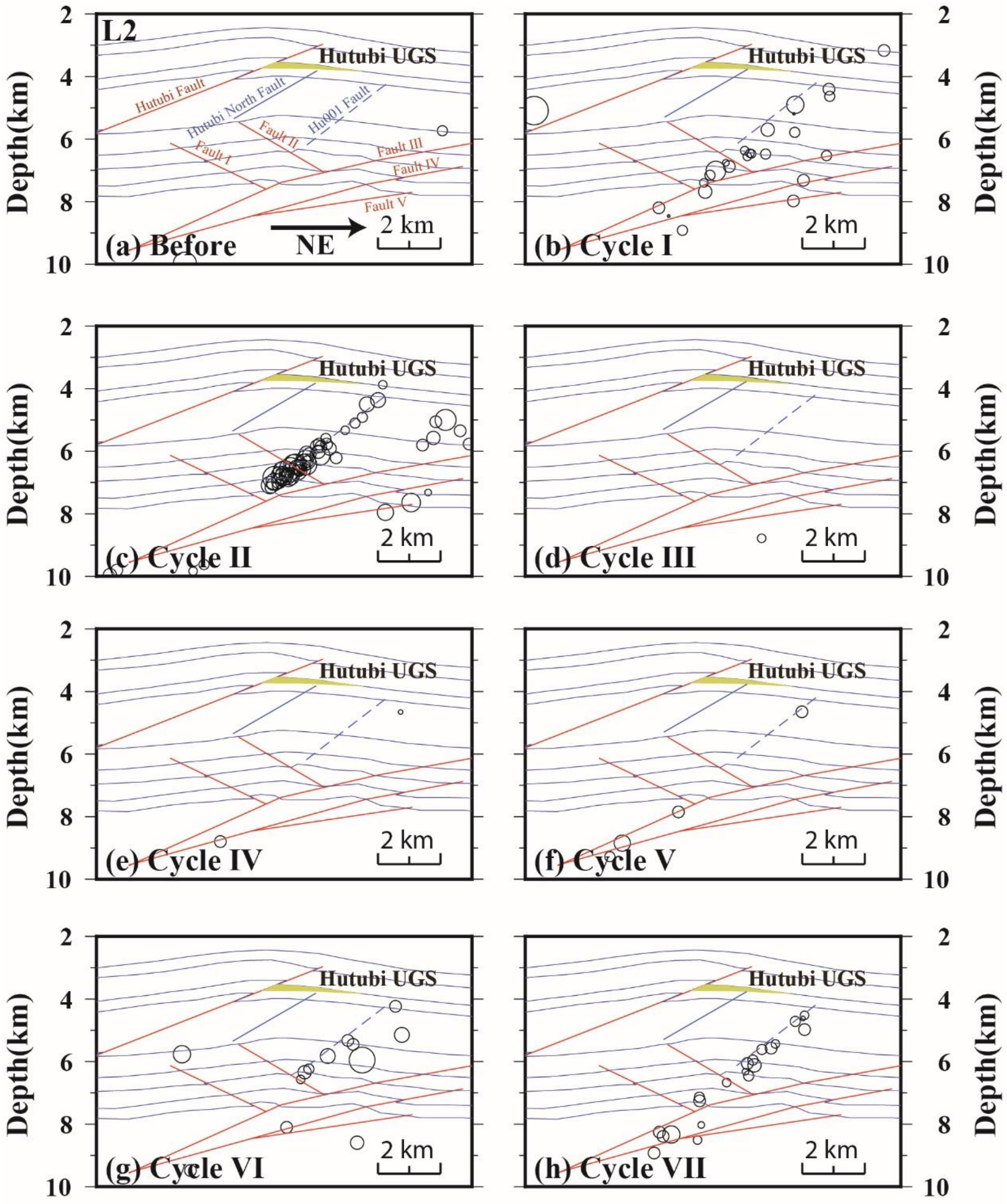

Figure S8. Section views along L2 (in Figure 5a) of the events in cluster β at different time periods. **(a)** Events before the UGS operation**. (b–h)** Events during each operation cycle. The events are denoted as circles scaled by their magnitudes. The strata (blue curve) and fault (red curve) data are from Jiang *et al.*[16]. The Hutubi North fault (blue solid line) and westerly extension of the Hu001 fault (blue dashed line) data are from Pang *et al.*[18]. The Hutubi UGS Reservoir is marked as the yellow area.

S8. Waveform similarity of two clusters

The waveform similarity between different clusters by calculated the waveform cross-correlations of P-waves of all event pairs in the two clusters at HTB station, which is the closest permanent station to the Hutubi UGS. The waveform is filtered to 2-8Hz, and the time window for the calculation is set to 2-s (0.5s before and 1.5 s after). A total of 181,836 event pairs from 450 relocated events were calculated. We counted the P-wave cross-correlations of event pairs within cluster α (1,554 in total), within cluster β (165,338 in total), and cluster α vs cluster β (14,944 in total), and the average value of them were 0.549, 0.508, and 0.404, respectively (Figure S9). Intra-cluster pairs have higher waveform similarity (with average cc>0.5) than inter-cluster pairs (with average cc~0.4).

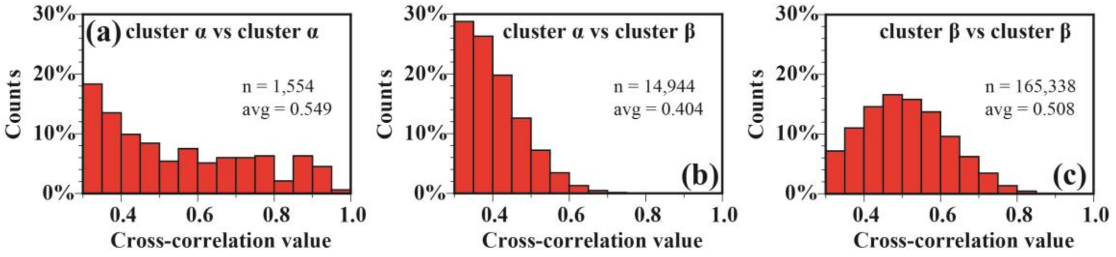

Figure S9. The P-wave cross-correlation of **(a)** cluster α vs cluster α, **(b)** cluster α vs cluster β, and **(c)** cluster α vs cluster β at HTB station.

S9. Epidemic-type aftershock sequence (ETAS) modeling

To quantitatively investigate the variation in multi-period operation seismicity, we fitted the seismicity data to the ETAS model, which is frequently used to separate the total seismicity into Omori-type aftershocks and external force-driven activity[10,11]. In the case of injection-induced seismicity, the external forces are the background tectonic loading and the injection-induced loadings[12].

We estimate the time-dependent forcing rate and other parameters following the method mentioned in Lei et al. (2013, 2017). Here we briefly describe its method, it consists of 4 steps: 1. Initially assuming a constant forcing rate $\lambda_0(t)=\lambda_0$; 2. Estimating the minimum AIC ETAS parameters $\theta(K, \alpha, c, p)$ knowing $\lambda_0(t)$; 3. Updating the estimate of the forcing rate based on these parameters; 4. Repeating steps 2 and 3 until all parameters converged. Model parameters were estimated by minimizing the Akaike information criterion (AIC)[12] (Figure S10).

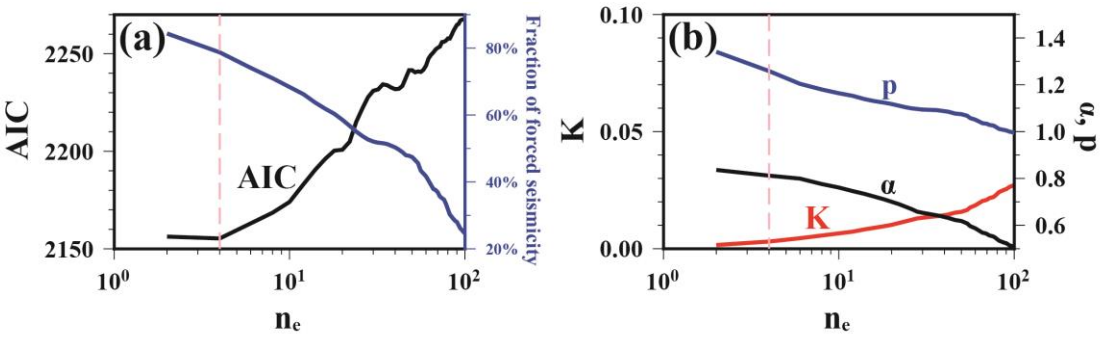

Figure S10. ETAS parameter K, α, p, and AIC, together with the total fraction of forcing seismicity. The minimum AIC is obtained for n_e=4.

We carried out the robust estimation of the standard errors of the ETAS parameters using Monte Carlo simulations. Following the 4 steps method mentioned in previous

study[12], 1,000 ETAS model simulations were run, and the ETAS parameters of each simulated earthquake sequence were estimated. For each run, ETAS parameter estimates of the simulated earthquake sequence were obtained. The standard error could then be estimated from these simulations by using the root mean square of the errors of the estimated parameters from the simulations as

$$SE = \sqrt{\sum_{i=1}^{N}(\theta_i - \theta_{ture})^2/N}, \quad \theta = \{\lambda_0(t), K, c, p\}, \tag{3}$$

Where θ_i and θ_{ture} are the estimated parameters from the ith simulation and the real data, respectively. The standard errors of the ETAS parameters (*f.s.*, K, α, p) shown in the Figure S11.

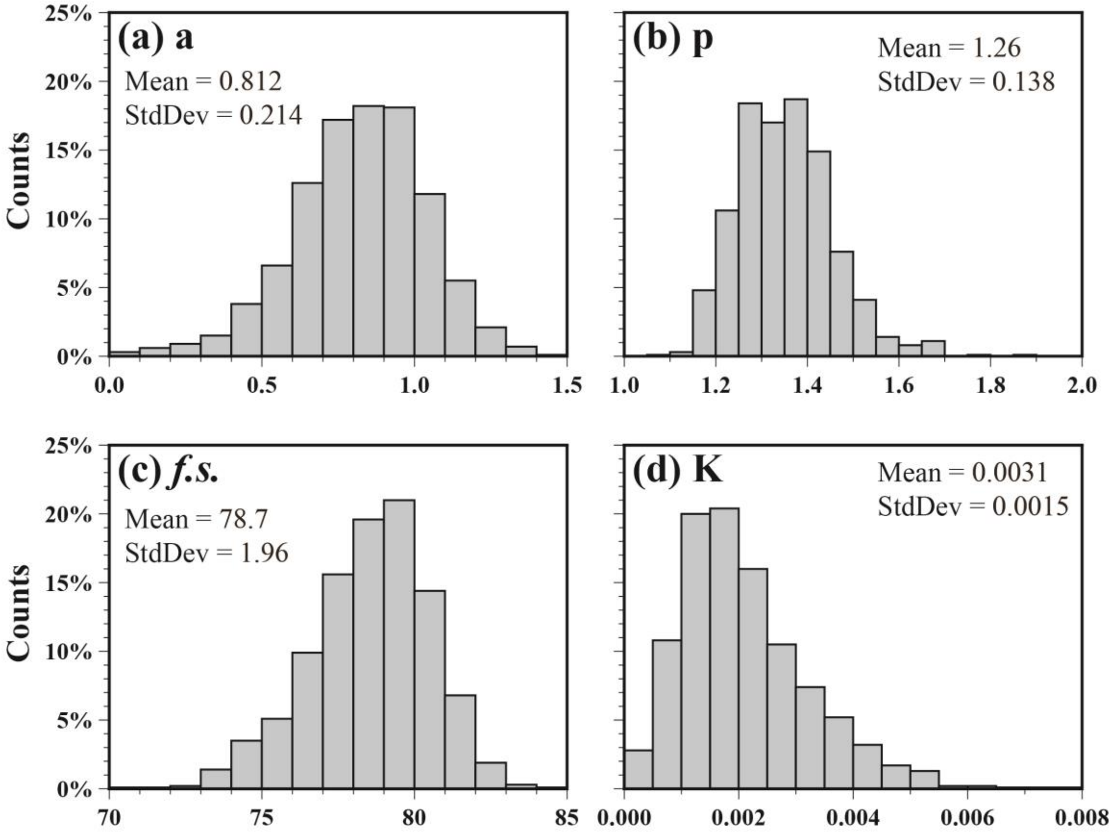

Figure S11. Results of Monte Carlo simulations for estimating the standard errors of the ETAS parameters (*f.s.*, K, α, p) shown in Figure S12. In total, 1,000 ETAS model

simulations were run, and the ETAS parameters of each simulated earthquake sequence were estimated.

When used with the time-varying forcing rate, the ETAS model demonstrates a total forcing rate of up to ~80% (79.9% in Figure S11) and shows that Omori-type aftershocks are rare, encompassing only 20% of all earthquakes. This suggests that the seismicity is likely governed by external forces[12,13]. This result is similar to seismicity induced by water injection[12,14].

After the Hutubi UGS operation began, the background forcing rate increased significantly. Two high background forcing rate were consistent with the first two injection periods (Figure S12), which indicates a relationship between the changes in seismic pattern and gas injection. The high background forcing rate further confirms that the seismic surges experienced in the first two injection periods were related to the UGS operation[1,2].

The seismicity rate quickly decreased to a much lower level following the two surges, similar to the ETAS modeling results seen for seismic activity relating to hydraulic fracturing[13]. In addition, the forcing rate gradually recovered when entering the stable operation stage (Figure S12).

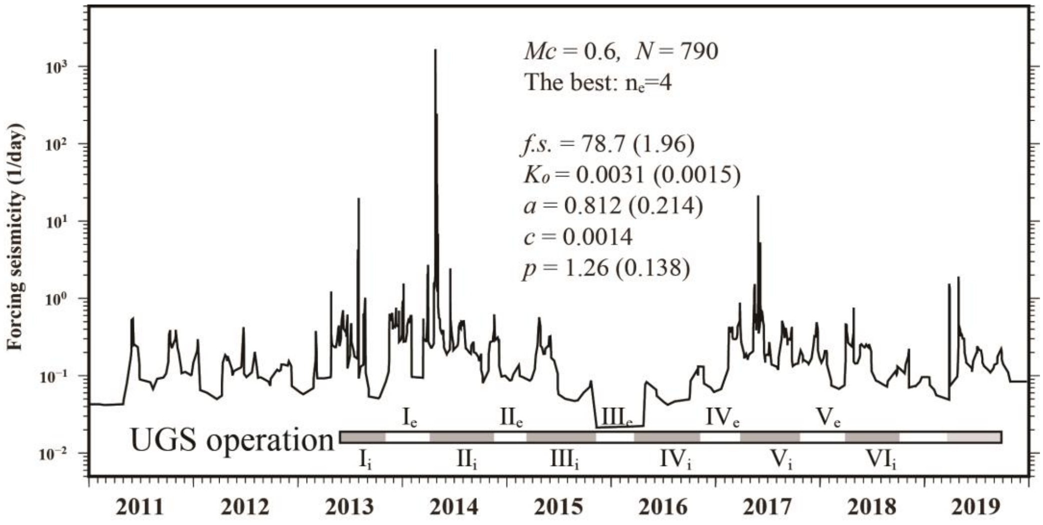

Figure S12. An epidemic-type aftershock sequence (ETAS) model for the detected seismicity. It shows the time-varying forcing rate (the black curve) and the ETAS parameters for the study's time window. The gray and white lines represent the injection and extraction stages, respectively.

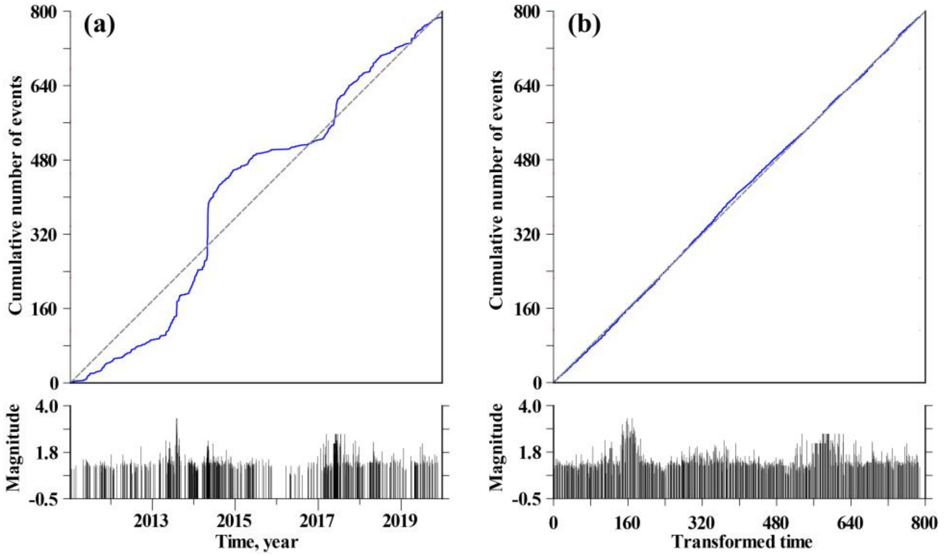

Figure S13. Cumulative number and magnitude of events against ordinary time **(a)** and transformed time **(b)** by the ETAS model from the Hutubi UGS. Grey dashed curves represent the theoretical cumulative numbers against the ordinary time and the transformed time, respectively.

S10. Comparison of earthquake locations from different studies

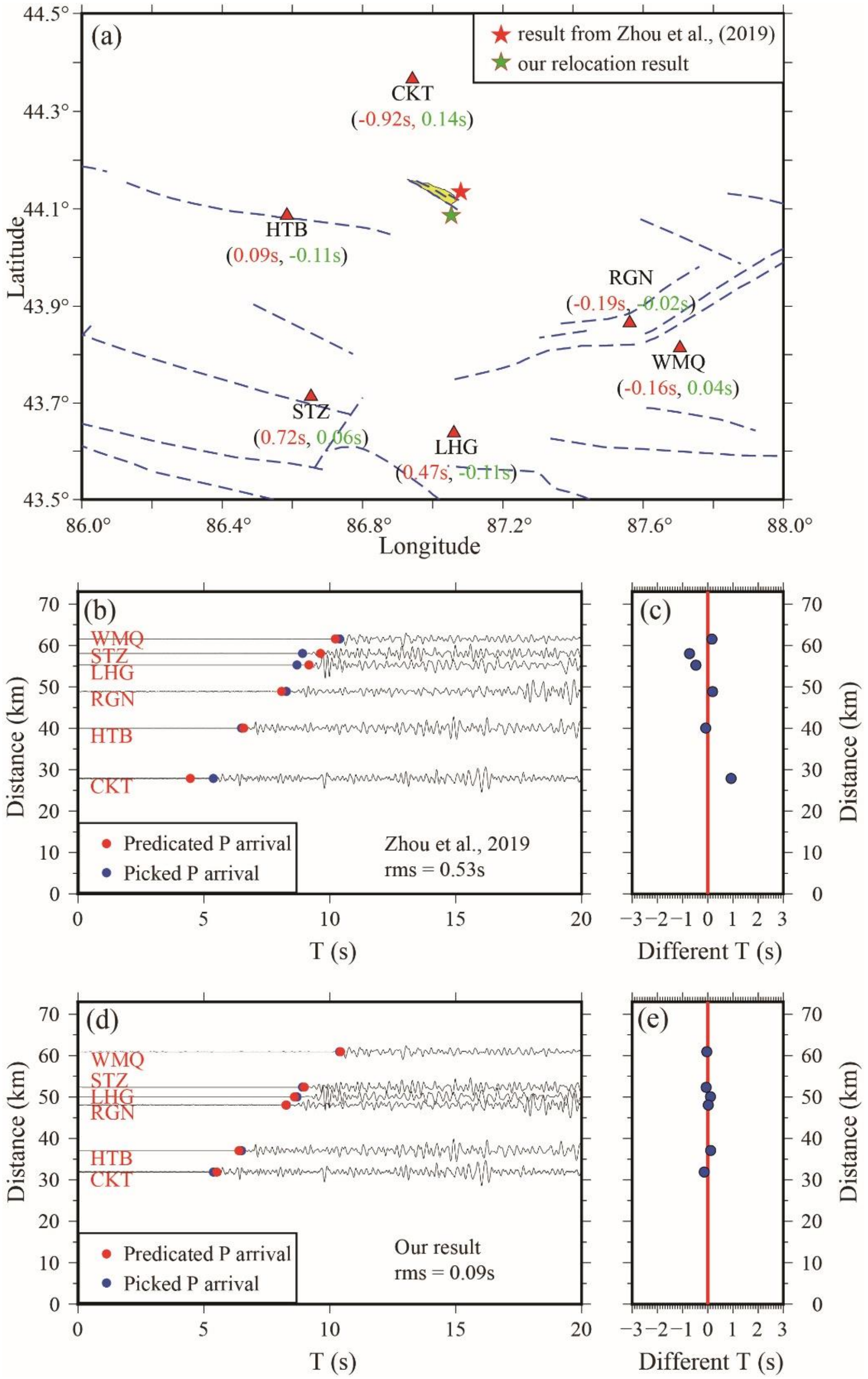

Figure S14. Comparison between different locations of the M_L3.1 event occurred on August 3, 2013. **(a)** The locations by Zhou *et al.*[2] and this study are marked as red and green stars, respectively. The P- wave travel-time residuals are labeled below the station name with their corresponding colors. Pseudo record sections and corresponding P-

wave travel time residuals according to the locations by Zhou *et al.*[2] **(b) & (c)** and this study **(d) & (f).** The red and blue dots illustrate the predicated and manually picked P-wave arrival times, respectively. The red dots denote the predicated P-wave arrival time, and the blue dots show the picked P-wave arrival time at each station.

S11. Estimating permeability

Permeability can be estimated by calculating hydraulic diffusivity using the Biot equation[15]:

$$D = Nk/\eta, \tag{4}$$

where D, k, and η are diffusivity, permeability, and pore-fluid dynamic viscosity, respectively. The N is a poroelastic modulus defined as $N = MP_d/H$; $\alpha = 1 - K_d/K_g$;$M = (\phi/K_f + (\alpha - \phi)/K_g)^{-1}$; and $H = P_d + \alpha^2 M$; $P_d = K_d + 4/3\mu_d$. Here K_d, K_f, and K_g are the bulk moduli of dry-frame, fluid, and grain material, respectively, μ_d is the shear modulus of frame material, and ϕ is the porosity. For the low-porosity layer, the terms of order α^2 can be neglected in comparison with terms of order 1 and α (in our case, $\alpha \approx 0.3$). The poroelastic modulus N is valid within the low-porosity layer and can be calculated using the following equation:

$$N = \left(\frac{\phi}{K_f} + \frac{\alpha - \phi}{K_g}\right)^{-1} \tag{5}$$

To estimate N, we used data from the model detailed by Jiang *et al.*[16], which agrees well with the observed ground extension and wellhead pressure data[16,17]. The parameters of the layers and faults in the model, such as the elastic moduli and the poroelasticity, are shown in Table S1. The permeabilities estimated from the diffusion coefficient of surge B in the two underlying layers (basal layers 1 and 2 in the geomechanical model) were 29.31 and 23.70 md, respectively.

Table S1. Mechanical and hydraulic parameters of the rock formations and faults in the geomechanical model for the Hutubi UGS

Layer	Density (Kg/m^3)	Yonug's modulus (GPa)	Biot coefficient	Poisson's ratio	Porosity (%)	Viscosity (Pa s)	Compressibility (Pa^{-1})
**Reservoir layer**	2362	24.37	0.39	0.31	3 ~ 30		
**Basal layer 1**	2394	28.68	0.32	0.30	5		
**Basal layer 2**	2488	44.09	0.09	0.27	5		
**Faults**	2488	10	0.79	0.2	5		
**Gas**	154					2.12×10^{-5}	1×10^{-5}
**Water**	1000					55×10^{-5}	0.42×10^{-9}

S12. Relocation result during the stable operation stage

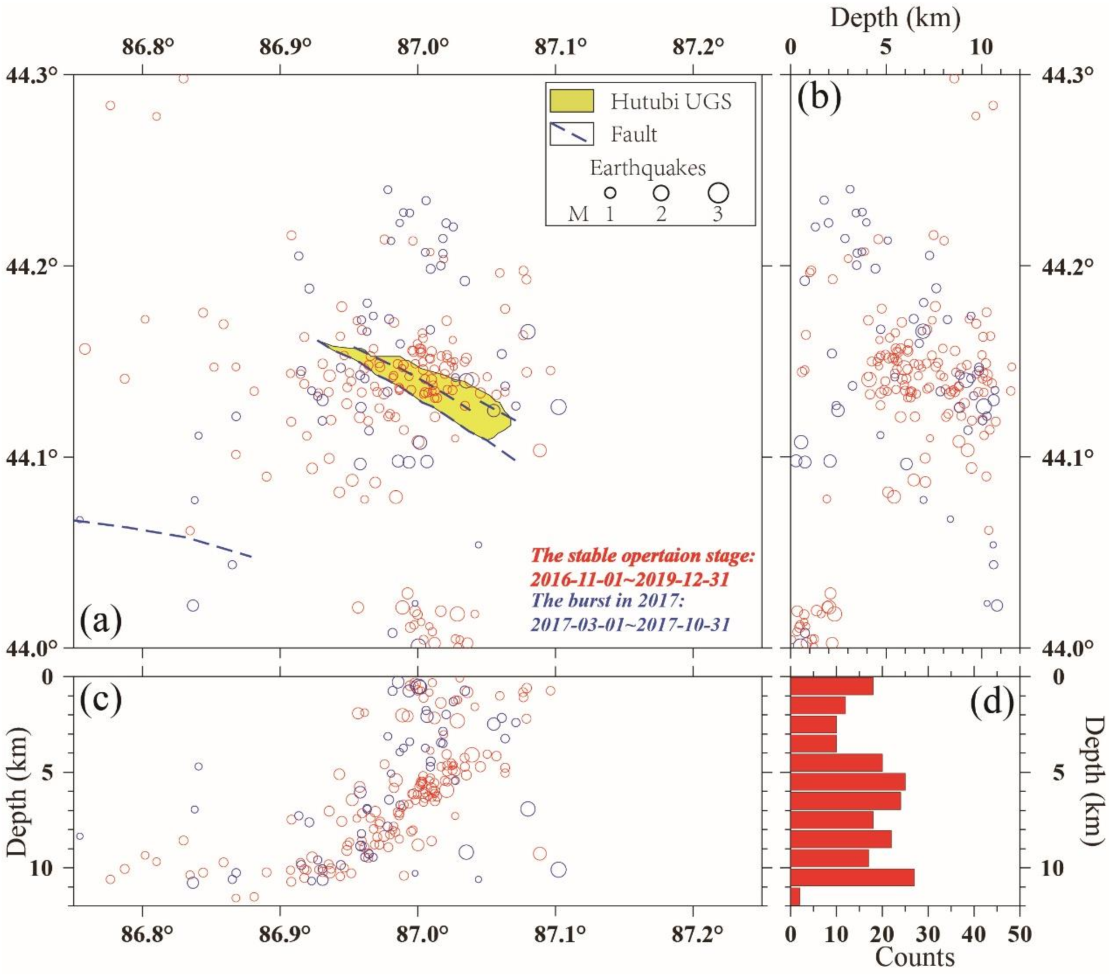

Figure S15. The map view of the relocated events in the stable operation stage. **(a–c)** All relocated events within 10 km of the Hutubi UGS are marked with circles scaled by their magnitude. The yellow area illustrates the Hutubi UGS. The depth distribution of the earthquakes is indicated by the red histograms in **(d)**.

Captions for Videos S1

Videos S1. The gas pressure diffusion in the reservoir and the seismicity during the second injection period. The gas pressure diffusion is the increment relative to the pressure state on April 6, 2014.

Supplementary Reference

1 Tang, L., Lu, Z., Zhang, M., Sun, L. & Wen, L. Seismicity Induced by Simultaneous Abrupt Changes of Injection Rate and Well Pressure in Hutubi Gas Field. *Journal of Geophysical Research: Solid Earth* **123**, 5929-5944, doi:10.1029/2018jb015863 (2018).

2 Zhou, P., Yang, H., Wang, B. & Zhuang, J. Seismological Investigations of Induced Earthquakes Near the Hutubi Underground Gas Storage Facility. *Journal of Geophysical Research: Solid Earth* **124**, 8753-8770, doi:10.1029/2019jb017360 (2019).

3 Klein, F. W. User's guide to HYPOINVERSE-2000, a Fortran program to solve for earthquake locations and magnitudes. Report No. 2002-171, 123 (2002).

4 Ji, Z. *et al.* Observation of Higher-Mode Surface Waves from an Active Source in the Hutubi Basin, Xinjiang, China. *Bulletin of the Seismological Society of America* **111**, 1181-1198, doi:10.1785/0120200272 (2021).

5 Waldhauser, F. A Double-Difference Earthquake Location Algorithm: Method and Application to the Northern Hayward Fault, California. *Bulletin of the Seismological Society of America* **90**, 1353-1368, doi:10.1785/0120000006 (2000).

6 Yang, H., Zhu, L. & Chu, R. Fault-Plane Determination of the 18 April 2008 Mount Carmel, Illinois, Earthquake by Detecting and Relocating Aftershocks. *Bulletin of the Seismological Society of America* **99**, 3413-3420, doi:10.1785/0120090038 (2009).

7 Richter, C. F. An instrumental earthquake magnitude scale. *Bull. Seismol. Soc. Am.* **25**, 1–32 (1935).

8 Wiemer, S. A Software Package to Analyze Seismicity: ZMAP. *Seismological Research Letters* **72**, 373-382, doi:10.1785/gssrl.72.3.373 (2001).

9 Wiemer, S. & Wyss, M. Minimum Magnitude of Completeness in Earthquake Catalogs: Examples from Alaska, the Western United States, and Japan. *Bulletin of the Seismological Society of America* **90**, 859-869, doi:10.1785/0119990114 (2000).

10 Ogata, Y. Statistical models for earthquake occurrences and residual analysis for point processes. *Journal of the American Statistical Association* **83**, 9-27 (1988).

11 Zhuang, J., Ogata, Y. & Vere-Jones, D. Stochastic Declustering of Space-Time Earthquake Occurrences. *Journal of the American Statistical Association* **97**, 369-380, doi:10.1198/016214502760046925 (2002).

12 Lei, X. *et al.* A detailed view of the injection-induced seismicity in a natural gas reservoir in Zigong, southwestern Sichuan Basin, China. *Journal of Geophysical Research: Solid Earth* **118**, 4296-4311, doi:10.1002/jgrb.50310 (2013).

13 Lei, X. *et al.* Fault reactivation and earthquakes with magnitudes of up to Mw4.7 induced by shale-gas hydraulic fracturing in Sichuan Basin, China. *Sci Rep* **7**, 7971, doi:10.1038/s41598-017-08557-y (2017).

14 Lei, X., Yu, G., Ma, S., Wen, X. & Wang, Q. Earthquakes induced by water injection at ~3 km depth within the Rongchang gas field, Chongqing, China. *Journal of Geophysical Research* **113**, B10310-B10310,

doi:10.1029/2008jb005604 (2008).
15 Biot, M. A. Mechanics of Deformation and Acoustic Propagation in Porous Media. *Journal of Applied Physics* **33**, 1482-1498, doi:10.1063/1.1728759 (1962).
16 Jiang, G. *et al.* GPS observed horizontal ground extension at the Hutubi (China) underground gas storage facility and its application to geomechanical modeling for induced seismicity. *Earth and Planetary Science Letters* **530**, 115943-115943, doi:10.1016/j.epsl.2019.115943 (2020).
17 Jiang, G., Liu, L., Barbour, A. J., Lu, R. & Yang, H. Physics-Based Evaluation of the Maximum Magnitude of Potential Earthquakes Induced by the Hutubi (China) Underground Gas Storage. *Journal of Geophysical Research: Solid Earth* **126**, 1-24, doi:10.1029/2020jb021379 (2021).
18 Pang, J. *et al.* Evaluation of sealing ability of underground gas storage converted from the Xinjiang H gas field. *Natural Gas Industry* **32**, 83, doi:10.3787/j.issn.1000-0976.2012.02.020 (2012).
