# Supplementary figures and images for: Spatiotemporal evolution of seismicity during the cyclic operation of the Hutubi underground gas storage, Xinjiang, China
Source: Sci Rep. 2022 Aug 24;12:14427. doi: 10.1038/s41598-022-18508-x (PMC9402545; doi:10.1038/s41598-022-18508-x)

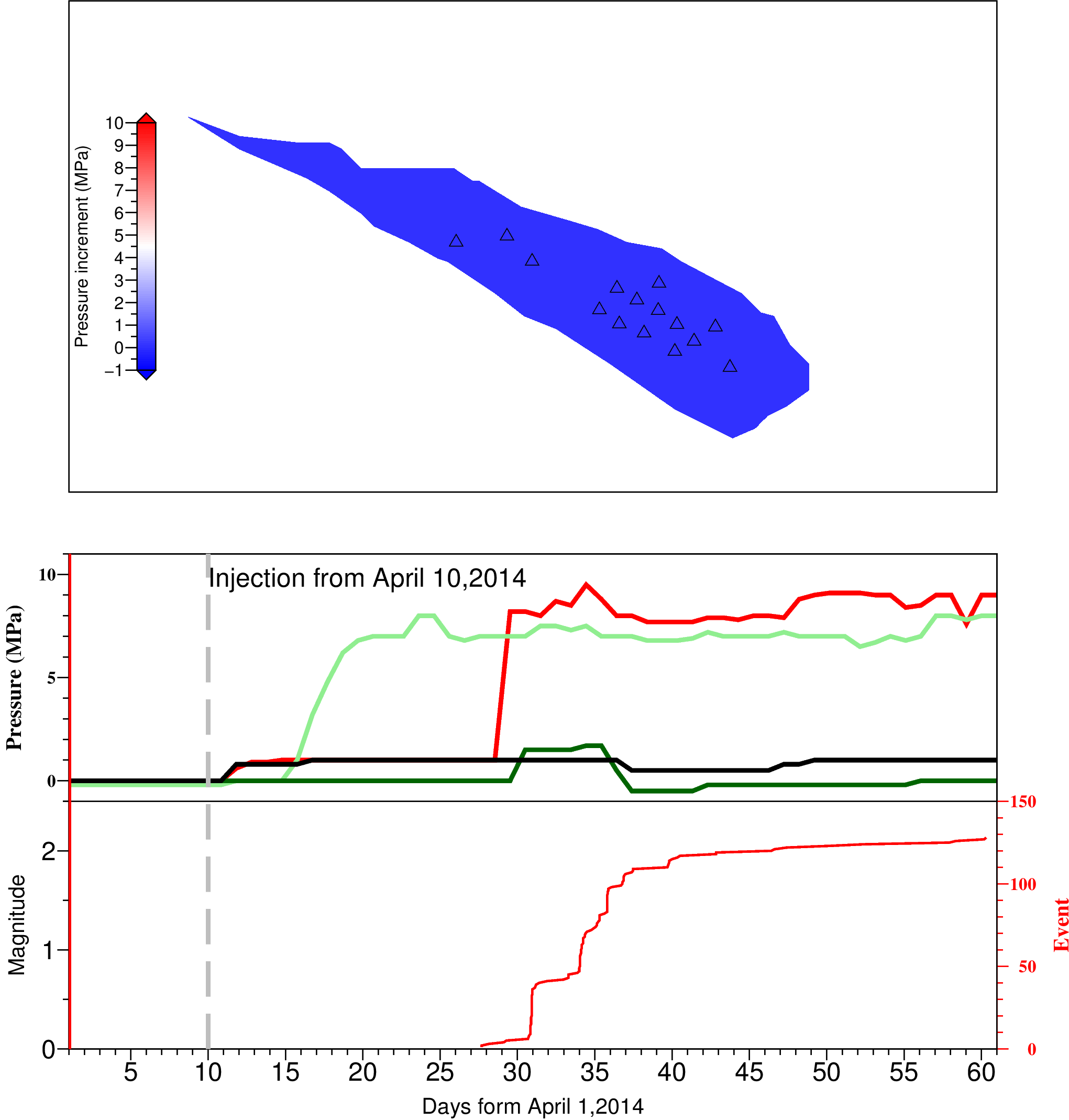

Supplement: Supplementary file 2 — Supplementary Information. [file 41598_2022_18508_MOESM2_ESM.gif]
